# Are students dependent on AI in writing courses? Analyzing factors influencing dependence on generative AI through the I-PACE model

**DOI:** 10.3389/fpsyg.2026.1905037

**Published:** 2026-09-11

**Authors:** Lili Liu, Meilian Zhuang, Jing Wang

**Affiliations:** School of Culture and Communication, Putian University, Putian, China

**Keywords:** academic writing, artificial intelligence, dependence, I-PACE model, students

## Abstract

**Background:**

The rapid penetration of artificial intelligence (AI) in the field of education has brought about an improvement in learning efficiency, but it has also triggered serious concerns. The harms of students' excessive dependence on AI to complete learning tasks, the weakening of critical thinking, and the degradation of self-learning ability are gradually emerging. This forces researchers to deeply understand the intrinsic mechanism of users' psychological dependence on AI. The theoretical framework of problematic behavior has been fully developed and can effectively explain and predict users' emotional connection, cognition, trust, and degree of dependence when using AI.

**Method:**

This study adopts a mixed-method design. For the quantitative phase, survey responses were collected from two universities in China. This component aims to validate the influence of AI dependence in the context of academic writing. For the qualitative phase, in-depth interviews were conducted with eight respondents. Quantitative data were analyzed using structural equation modeling, while qualitative data were processed through thematic analysis. This study explores Chinese college students' motivations for excessive AI use and their perceptions of AI dependence.

**Results:**

Quantitative research indicates that academic stress, AI literacy, and perceived trust have a significant impact on AI dependence, while perceived usefulness can mediate the relationship between academic self-efficacy and academic stress. Perceived trust plays a mediating role between social influence and AI dependence. Social influence predicts AI dependence behavior through perceived trust. Qualitative research indicates that although students are aware of the risks of AI, factors such as academic stress, peer influence, the efficiency of tools, and policy ambiguity are significant incentives for the strategic use of AI; there exists a strong psychological connection with AI dependence.

**Discussion:**

The study extends the I-PACE model to generative AI academic writing contexts, revealing the specific social impact and the trust transmission mechanism. The study also emphasized that academic stress and AI literacy are important influencing factors. For educational practice, both protective factors and risk factors should be taken into account: efforts should be made to enhance students' AI literacy, while also paying attention to alleviating excessive academic stress on students, in order to reduce the excessive dependence of college students on generative AI. This study emphasizes that responsible use of AI should be promoted in the process of introducing AI into teaching.

## 1 Introduction

Generative artificial intelligence (AI) applications in the field of education have revolutionized teaching methods, particularly profoundly changing the way college students engage in academic writing. Generative AI tools are feature-rich: they can generate creativity, write content, check grammar, and even write complete papers (Giray et al., 2025). Learners can use generative AI to handle mechanical tasks such as information retrieval, data organization, and language refinement, thereby concentrating their academic time on higher-level critical thinking, creative generation, and more complex problem-solving. As AI continues to evolve, its role in education is expanding, and it has become an indispensable tool in modern teaching and learning.

For students facing writing difficulties or time constraints, this technology seems to provide them with a fair and competitive environment (Kim et al., 2024). However, this convenience has triggered a more complex ethical dilemma. Some students gradually transition from using AI to correct grammatical errors and polish their writing to depending on AI to conceive writing frameworks and generate complete paragraphs, submitting AI-generated content as original work, which circumvents the core elements of cognition and reflection in academic research (Darvishi et al., 2024). This shift not only gives rise to new forms of academic misconduct but also raises concerns about learners' excessive dependence on AI in academic writing (Bower et al., 2024). Generative AI can improve writing efficiency, enhance writing quality, and reduce writing anxiety, but habitual use over a long period may lead to cognitive offloading. This phenomenon manifests as the weakening and replacement of learners' thinking abilities, a decrease in intrinsic learning motivation and autonomous learning ability, and direct dependence on AI for generating content and insights when academic difficulties arise (Celik et al., 2022). In the long run, under certain conditions, continuous functional use may gradually develop into an maladaptive dependence state, characterized by an inability to detach from AI tools, uncritically accepting AI outputs, and a significant decline in autonomy in the learning and decision-making process, and an excessive dependence on AI-generated content (Acosta-Enriquez et al., 2025).

It is noteworthy that, despite the increasing attention to generative AI in the education field, the existing academic research on generative AI dependence in educational contexts is still insufficient (Meng et al., 2022). This phenomenon cannot be simply attributed to convenience or laziness issues, but should focus on the psychological and social driving factors of problem behaviors. We must delve into the psychological mechanisms of students' AI dependence in an academic environment, such as which groups are more susceptible, which emotional and cognitive factors are related to this dependence, whether there are factors that exacerbate or mitigate these relationships, and how these factors are associated with each other. We believe that the answers to these questions are crucial for the rational application of generative AI in education.

To address these concerns, this study employs the I-PACE model (Interaction of Person-Affect-Cognition-Execution) to explore the intrinsic mechanism of excessive dependence on generative AI among university students in academic writing (Brand et al., 2016). Because the model framework can fully capture the maladaptive dynamics surrounding AI from the perspectives of personal traits, emotions, and cognition, The I-PACE framework indicates that personal traits determine susceptibility, while the cognitive and emotional responses of individuals to internal and external stimuli, combined with the continuous strengthening of executive functions, collectively maintain long-term habitual use behaviors (Brand et al., 2016). This study aims to reveal the potential mechanisms of AI dependence among university students in academic writing by analyzing personal trait factors such as academic self-efficacy, AI literacy, academic stress, perceived trust, social influence, and perceived usefulness, as well as emotional and cognitive factors. To gain a deeper understanding of excessive dependence behavior, this study also considers it through open-ended questionnaires and interviews. The results of this study will provide important insights into the mechanisms and driving factors of AI dependence in the educational field, and guide university students on how to use AI responsibly in academic writing without over-reliance. The research findings will also provide feasible strategy references for educational management institutions and policy-makers, to support teachers in understanding the learning habits, decision-making methods, and participation in the long-term use of AI tools among university students, and help teachers reasonably introduce AI technology and effectively maintain students' digital mental and physical health in this process.

## 2 Literature review

### 2.1 Generative AI dependence

With the increasing maturity of generative AI technology and its wide application, research on problematic behavior and dependence on AI is in a gradual upward phase. Existing literature on the use of AI has used different terms to describe it, including “dependence,” “problematic use,” and “addiction.” Although there is no unified conceptual definition in the academic community, after sorting out the boundaries between concepts, (Dagnino et al., 2026) proposes that “problematic use behavior” is the most comprehensive concept among the three, with its core concern being the functional impairment and consequences caused by the use of generative AI. It focuses on the consequences of behavior, including declining grades, violations of integrity, and other related issues, without involving clinical diagnosis and pathologizing tendencies. “Addictive behavior” generally refers to symptomatology with clinical significance, including elements such as craving, physiological tolerance, and pathological impairment of executive functions. “Dependence” represents a form of problematic use. It focuses on the voluntary surrender of independent cognitive and psychological engagement. For instance, individuals offload core cognitive functions such as critical thinking and decision-making to AI, leading to a degradation of their own capabilitie (Kong et al., 2026).

Generative AI dependence has been discussed in relation to cognitive offloading across multiple studies. According to the cognitive offloading theory (Risko and Gilbert, 2016), due to the limitation of internal memory capacity, individuals tend to outsource high-level cognitive tasks such as memory and reasoning to external tools, thereby releasing internal cognitive resources. Moderate cognitive offloading can improve writing efficiency, but long-term excessive outsourcing of core thinking tasks shows maladaptive cognitive offloading. Repeated cognitive offloading will promote the formation of cognitive dependence, specifically manifested as the psychological tendency of individuals to outsource their thinking to AI when facing tasks. This constitutes a “dependence paradox”: students continue to use AI to alleviate cognitive burden, but gradually lose their independent writing and thinking abilities, and develop a stable dependence on AI tools (Park et al., 2025). AI has the potential to efficiently complete tasks and promote emotional connections, gradually leading to a subtle problematic use pattern. It is a habitual delegation of core cognitive functions to tools, which constitutes a maladaptive cognitive offloading (Dagnino et al., 2026). In this research context, “Generative AI dependence” refers to students' habitual use of AI in academic writing, developing into a maladaptive cognitive offloading, and ultimately forming a psychological mechanism of excessive dependence on AI.

Some studies suggest that AI dependence involves both cognitive dependence and emotional dependence. Cognitive dependence refers to the situation where individuals entrust their mental activities, such as thinking and reasoning to AI. Emotional dependence, on the other hand, refers to the way individuals rely on AI to alleviate stress and negative emotions (Wu et al., 2026). The research indicates that cognitive (over) dependence is associated with performance expectations, demand frustration, and trust in AI, while psychological and emotional reliance is linked to feelings of loneliness, anxiety, anthropomorphism tendencies, and reliability perception (Dagnino et al., 2026). From a psychological perspective, factors such as stress, anxiety, and the cognitive perception of the practicality of AI may contribute to the formation of AI-dependent behaviors (Huang et al., 2024). People who feel lonely may cope with social difficulties and alleviate emotions by interacting with AI, thereby exacerbating the degree of dependence. The convenience of AI-generated content, combined with immediate feedback and adaptive learning mechanisms, easily reinforces continuous use behaviors, forming a habitual cycle (Renshaw and Carley, 2024). Learners who maintain close cognitive and emotional connections with AI for a long time may gradually find it difficult to independently regulate their use behaviors, eventually developing into excessive dependence or even addiction (Dinnie, 2024). In the field of education, if college students over-rely on AI, they may experience a decline in creativity, difficulty in completing tasks without AI support, or directly copying and using content generated by AI to complete academic tasks.

Some studies believe that there are certain similarities between generative AI dependence and previous internet addiction and social media addiction, such as: the practicality and pleasure brought by media or technology may trigger habitual excessive use phenomena and addictive behaviors, typical manifestations include excessive use time, difficulty in stopping use, anxiety after interruption, dependence on AI for decision-making, and the regression of self-problem-solving abilities. The efficiency and convenience brought by AI tools may trigger a desire and repetitive use cycle similar to social media addiction.

### 2.2 I-PACE model

In 2016, scholars Brand proposed the I-PACE model (Interaction of Person-Affect-Cognition-Execution) to explain the causes of specific Internet-use disorders (Brand et al., 2016). The I-PACE model provides a theoretical framework for understanding addictive use behaviors, elucidating the mechanisms underlying addiction to internet applications that facilitate behaviors such as gaming, gambling, pornography viewing, and online shopping. The model emphasizes that the occurrence and development of addictive behaviors are the result of the interaction between users' emotional and cognitive responses to specific situational stimuli and their executive functions. The P component of the model points to user characteristics such as psychopathological features, individual personality, and social cognition; the A component and the C component represent the emotional and cognitive responses produced after specific situational stimuli, such as coping strategies, cognitive biases related to the internet, attentional biases, and emotional regulation; these emotional and cognitive responses lead to changes in the E component of the model, such as problems with executive functions or decreased inhibitory control. Ultimately, these components interact and have a synergistic or partial impact on the occurrence and development of addiction.

I-PACE theory provides an insightful model framework for scholars concerned with internet addiction. Based on this model, past research has analyzed digital technology addiction in areas such as social media, smartphones, and online games. The latest revised version of the model further supports its application in various fields. Fortunately, although research on problematic behavior related to AI dependence is still in its early stages in the context of applying generative AI in education, people are beginning to realize that the I-PACE model can provide an important theoretical perspective for studying college students' dependence on generative AI. Zhang et al. (2024) is one of the earliest studies to explore the internal antecedents and potential consequences of AI dependence using the I-PACE model. The I-PACE model explains the process of technology dependence primarily through three components. Personal traits, such as academic self-efficacy, shape users' motivation and engagement in using generative AI tools. People with strong social anxiety and loneliness tend to repeatedly think about their negative experiences, and they may over-rely on AI as an adaptive coping strategy (Hu et al., 2023). At the emotional and cognitive levels, emotions, stress, and perceived usefulness, will further enhance the use of technology. Academic stress, performance expectations, and desire thinking are also somewhat related to generative AI dependence; those who expect AI to significantly improve academic performance may become increasingly dependent on it to cope with academic stress (Liu et al., 2026). AI can assist teachers in a series of tasks such as curriculum design, teaching evaluation, and administrative affairs. When teachers use these functions to reduce stress and improve efficiency, they receive positive emotional feedback, which further deepens their use of AI (Bower et al., 2024). In the long run, the combined emotional and cognitive effects may lead to a self-reinforcing cycle: AI promotes problem-solving, and interactions with AI seem more relaxed and efficient, but it subtly weakens the user's ability to self-regulate, thereby increasing their dependence on technological support. Teachers who frequently seek help from AI may gradually evolve from functional use to habitual use, eventually forming addictive dependence behaviors (Comiskey, 2024).

The I-PACE model provides an ideal theoretical framework for this study. This study has constructed a complete causal path from individual traits to the formation of AI dependence behaviors: the “Person” component represents the pre-existing influencing factors in the academic context, covering individual abilities, comprehensive qualities, and external environmental conditions, which are the fundamental prerequisites for triggering behavioral differences. The “Emotion and Cognition” component is jointly represented by academic stress, perceived trust, and performance expectations. These factors continuously shape users' emotional experiences and cognitive motivations toward generative AI. The “Execution” component serves as the outcome variable, referring to the excessive reliance on generative AI, encompassing an individual's cognitive reliance, emotional reliance, and tool reliance on AI tools. These aim to explain the mechanism by which individual trait factors gradually evolve into AI dependence.

### 2.3 Academic self-efficacy

Self-efficacy, this concept originates from the social cognitive theory (Bandura, 1986), refers to an individual's judgment and confidence in their ability to complete a certain task. Academic self-efficacy refers to an individual's belief and judgment about whether they can achieve a certain learning goal, which is the concretization of self-efficacy in the field of learning. In the I-PACE model, self-efficacy has been identified as a crucial variable in the “individual characteristics” dimension, and related research often explores the pathways of self-efficacy within the I-PACE framework, such as how self-efficacy affects the interaction between learners and platforms, peers, and teachers, thereby influencing their cognitive strategies, emotional engagement, and ultimately their learning performance.

Academic self-efficacy is a potential risk factor for AI dependence. Learners with lower academic self-efficacy may face higher academic stress; have insufficient confidence in learning, and weaker learning motivation. When encountering more difficult academic tasks, they tend to seek external support to avoid possible failure experiences. AI can help them improve their academic performance in the short term through its functions such as immediate feedback, task decomposition, non-judgmental interaction, and personalized assistance (Rahman and Watanobe, 2023). In the long term, students with lower academic self-efficacy may develop instrumental and psychological dependence on AI, leading to the emergence of excessive dependence behaviors. Based on this, we propose the following hypothesis:

**H1**: Academic self-efficacy has a negative impact on academic stress.

### 2.4 Academic stress

Academic stress is a persistent psychological load experienced by students in the pursuit of academic goals (Bedewy and Gabriel, 2015), which may trigger psychological and behavioral adaptation problems (Reddy et al., 2018). Within the I-PACE model framework, academic stress is considered a social cognitive factor, belonging to variable A in the model, and is an important trigger for internet use disorders. Those with a susceptibility to stress and impulsive coping strategies are more likely to experience impulsive regulation of emotions when faced with stressful situations. This psychological interaction may lead them to develop the expectation that “going online can relieve stress” or hold other cognitive biases related to the internet, thus making them more inclined to use the selected internet applications or websites.

Based on the stress coping theory (Lazarus and Folkman, 1984), individuals in stressful situations will actively seek ways to cope with external challenges and internal disturbances. For students facing high academic stress, AI technology, due to its convenience and rapid response capabilities, can efficiently synthesize and process academic information, thereby meeting academic needs in the short term and alleviating stress (Rani et al., 2023). Studies have shown that AI tools such as chatbots can also alleviate users' emotional stress through interaction, thereby improving their mental health status (Park et al., 2019). However, if the coping mechanisms adopted by individuals lack effectiveness, they may increase the probability of maladaptive or addictive behaviors (Metzger et al., 2017). The immediacy and personalized support provided by AI may induce excessive dependence in some individuals. Students with high academic stress are more inclined to seek academic and emotional support through A (Rani et al., 2023), while highly reflective individuals are more likely to use technology as a strategy to avoid stress. Based on this, we believe that learners with high academic stress may increasingly turn to AI as the main coping mechanism, thereby exacerbating their over-reliance on the technology. Therefore, we propose the following hypothesis:

**H2**: Academic stress has a positive impact on generative AI dependence.

### 2.5 Artificial intelligence literacy

AI literacy, as an emerging construct, has not yet formed a unified definition in the academic community. From the theoretical context, AI literacy originates from the theory of media information literacy (MIL) and is an extension of media information literacy and digital literacy in the intelligent era. This study adopts the definition currently cited most frequently: it refers to the comprehensive ability of individuals to understand the operation mechanism, application methods, and social impact of AI technology at the cognitive, evaluation, and practical levels (Long and Magerko, 2020). The United Nations Educational, Scientific and Cultural Organization (Miao et al., 2023) explicitly proposes in its policy brief that MIL is a core countermeasure against the risks of generative AI. MIL cultivates critical information evaluation abilities and incorporates cognitive requirements related to interpretable AI, empowering users to resist information manipulation and algorithmic bias triggered by AI. Enhancing the public's AI literacy is widely regarded as an important approach to addressing the challenges posed by AI-generated content (Ling, 2025). Relevant theories suggest that higher AI literacy enables the audience to possess stronger critical thinking skills, thereby more acutely identifying algorithmic biases and misinformation in AI-generated answers and further promoting more cautious and in-depth cognitive processing of the generated results (Deuze and Beckett, 2022). Previous research has confirmed that students with higher AI literacy demonstrate stronger abilities to discern the value of ChatGPT. An empirical study on the current status of generative AI dependence among college students found, when analyzing the relationship among basic psychological needs satisfaction, AI literacy, and academic burnout, that college students' AI literacy is negatively correlated with generative AI dependence (Guo and Li, 2026).

In fact, the relationship between AI literacy and technology may be more complex. Students with higher AI literacy can maintain a psychological distance from AI and control it, they are more likely to identify the accuracy of AI-generated content and prevent themselves from slipping from reasonable use to abuse. Empirical research shows that improving AI literacy can weaken the association between the motivation to use and problematic use of AI (Kong et al., 2026). Conversely, we believe that individuals with higher digital literacy are better able to critically evaluate AI outputs, strategically using the tool by determining its usefulness, and maintaining their subjectivity and responsibility in the learning process. Based on this, we propose the following hypotheses:

**H3**: AI literacy has a negative impact on generative AI dependence.

### 2.6 Social influence

Social influence refers to the extent to which individuals perceive that people who are important to them or have an impact on them believe that they should use new information technology. Social influence holds that psychological factors such as conformity, obedience, and imitation make people consciously or unconsciously conform to the norms of the majority or the group, ultimately affecting their attitudes and decision-making on behavior (Venkatesh et al., 2003). Social influence is also an important variable in the integrative technology acceptance and use theory model, which believes that the norms and behaviors of the group or important individuals directly affect people's behavioral decisions on information technology. According to the I-PACE model theory, the internal incentives for internet use (such as individual characteristics) and external incentives (such as everyone around them using it) will prompt individuals to develop emotional and cognitive responses to internet us (Brand et al., 2016). Within the I-PACE model framework, social influence is considered a social cognitive factor and an important trigger for network use barriers.

A large number of studies have confirmed that social influence has a significant impact on social media addiction and smart device addiction. For example, subjective norms and social identity have a significant predictive effect on the addictive use of smartphones (Chen, 2020). In short, people tend to follow the norms of their friends and family or the groups in schools/workplaces (Borsari and Carey, 2003). Peer effects play a crucial and indispensable role in the acceptance and use of AI by students. Encouragement and demonstration behaviors among peers can usually effectively mobilize the enthusiasm of the group, while some teachers, due to the failure to provide clear guidance, inadvertently further promote the spread of this effect (Giray et al., 2025). For example, the adoption of technology may be influenced by the consumption content of other group members (Ho et al., 2017). Therefore, this study believes that if people around recognize or frequently use AI, their willingness to use it may increase; the promotion of AI by school departments or teachers may encourage students to increase their use; and when their classmates commonly use AI to assist with homework, individuals may imitate to avoid isolation, which may lead to excessive use. Therefore, the following hypothesis is proposed:

**H4**: Social influence has a positive impact on generative AI dependence.

### 2.7 Perceived trust

Perceived trust is a psychological state where one has positive expectations regarding the intentions or behaviors of others and does not guard against them (Rousseau, 1998). In the field of AI, trust refers to the attitude of a subject in an unstable or vulnerable environment who believes that the agent can assist them in completing a specific task (Hoff and Bashir, 2015). McAllister distinguishes trust into cognitive trust and emotional trust. Within the I-PACE model, cognitive beliefs are manifested as an individual's level of trust in an internet platform, content, or other users, which influences their cognitive assessment (Jiayi, 2024). Excessive trust in or reliance on the internet may lead to cognitive biases, thereby promoting problematic internet use. Emotional trust is manifested in the ability of internet platforms to provide users with an emotional comfort zone, reduce anxiety and loneliness, and thereby facilitate individuals' emotional regulation. This emotional trust may encourage individuals to repeatedly engage in specific internet use behaviors such as prolonged socializing, gaming, and shopping, and may ultimately lead to problematic behaviors. Therefore, trust is not only a cognitive response to external or internal stimuli but also an emotional response and should be incorporated into the A and C components of the model.

According to the framework of automation trust and dependence, Trust is a prerequisite for the formation of reliance behavior, and the level of trust that users have in the automation system directly determines the extent to which they are willing to delegate tasks to the system. The empirical study supports this view; mathematics teachers with high trust in AI have a significantly higher degree of AI reliance than those with low trust (Wijaya et al., 2024). Perceived trust is a key antecedent to deepening AI dependence, as high trust reduces users' cognitive vigilance toward AI. The empirical research by Xie et al. (2023) on social chatbots found that users' trust in AI significantly and positively predicts their degree of AI dependence, indicating that trust is a key cognitive antecedent driving users' deep connection with AI and forming dependence. Existing research has confirmed that excessive trust can lead students to become overly dependent on generative AI. AI may also make mistakes or suffer from algorithmic bias, so blind trust in technology can bring risks because users may follow harmful advice, leading to adverse consequences. Therefore, this study believes that higher perceived trust will reduce students' cognitive vigilance toward AI, foster excessive emotional attachment, and ultimately lead to AI dependence. Based on this, the following hypothesis is proposed:

**H5:** Perceived trust has a positive impact on generative AI dependence.

We also believe that social influence brings another cognitive consequence: social influence enhances individuals' perceived trust in generative AI. This concept originates from social identity theory, where group identity prompts individuals to make consistent judgments, thereby enhancing perceived trust. The credibility of cues within the group is significantly higher than that of strangers. In terms of empirical research, Metzger et al. (2010) systematically discusses how heuristic cues from peer reviews and group consensus in online credibility assessment significantly and positively predict user information trust, especially when information is ambiguous. Existing empirical studies have shown that social norms have a significant positive predictive effect on individuals' rapid trust in AI, but the strength of the effect may vary due to cultural backgrounds. This finding provides cross-cultural evidence for the applicability of social influence theory in the field of AI trust (Etin et al., 2025). Therefore, this study believes that when surrounding peers or groups have a higher recognition and use AI more frequently, individuals are more likely to form stronger perceived trust. Social influence may not only directly affect AI dependence but is even more importantly, it may first shape users' psychological cognitive state of perceived trust, thereby indirectly catalyzing users' dependence behavior. That is, perceived trust plays a key mediating role in the relationship between “social influence” and “AI dependence.” Based on this, the following hypothesis is proposed:

**H6**: Social influence has a positive effect on perceived trust.
**H7**: Perceived trust mediates the relationship between social influence and generative AI dependence.

### 2.8 Perceived usefulness

Perceived usefulness is the individual's perception of the degree to which using a system can enhance their work performance, and it is a decisive factor affecting the individual's decision to accept and use a technological system (Davis, 1989). Brand proposes that expectations and related expectations for the use of the internet, often together with other cognitive illusions, are collectively referred to as internet-related cognitive biases. Internet use expectations may be an important mediating or moderating variable, potentially influencing the relationship between susceptible variables and technology use. In the adoption model of generative AI, perceived usefulness reflects the user's expectations of the effectiveness of the technological tool and essentially plays a regulatory cognitive bias role. It not only connects the two antecedent variables of academic self-efficacy and academic stress but also regulates the strength of their association with AI dependence (Zhang et al., 2024). Research shows that students can effectively alleviate anxiety and enhance the sense of control over the writing process by using generative AI for planning, drafting, and revising (De Silva, 2015). Therefore, students with lower academic self-efficacy are more likely to have excessive expectations and cognitive illusions toward technology, and consequently, to use excessive AI as a coping strategy. Based on these insights, we propose the following hypotheses:

**H8**: Perceived usefulness moderates the relationship between academic self-efficacy and academic stress.

**Figure 1:**
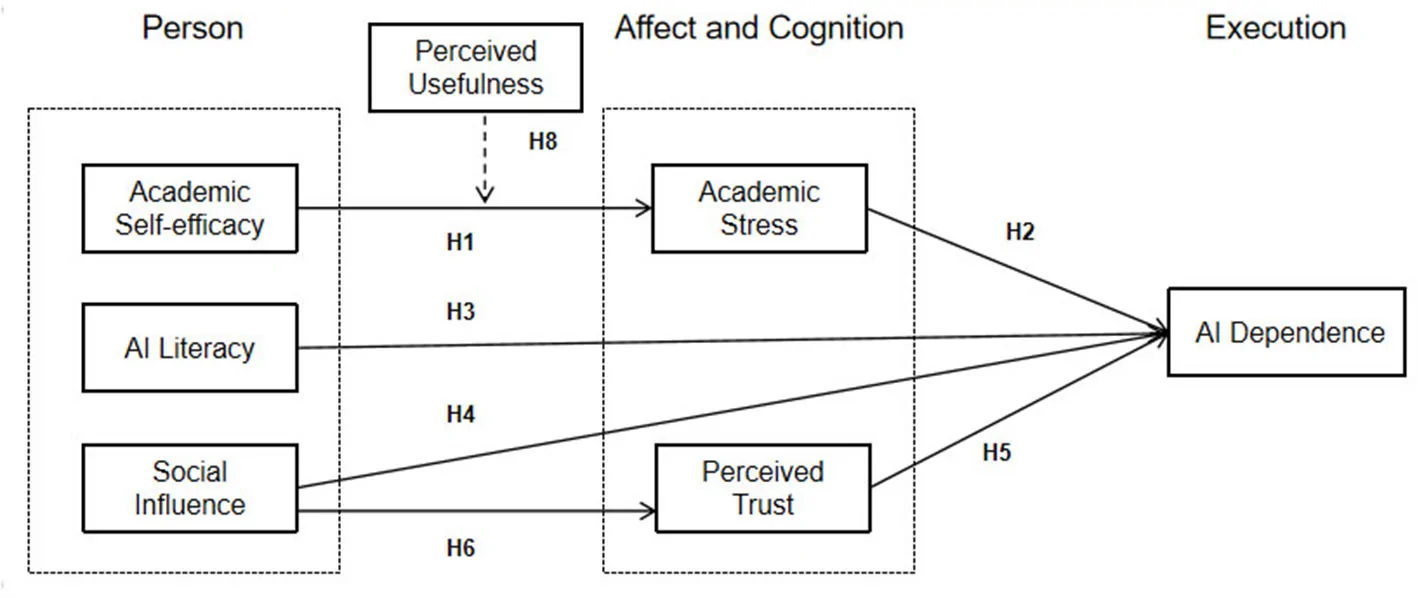
The proposed model.

This study theoretically integrates multiple theories in the I-PACE model, Figure 1 shows the final conceptual framework of this study, which clarifies the hypothetical relationships among various variables, how variables in personal traits, emotional factors, and cognitive factors interact to form AI dependence. Through the application of the I-PACE model, this study fills the research gap regarding generative AI dependence in college students' writing tasks and focuses specifically on the psychological mechanisms and contributing factors of dependence. To this end, this study aims to explore the following core issues:

RQ1: What factors predict college students' dependence on generative AI in academic writing?
RQ2: What are the motivations of students for using generative AI tools in academic writing, and how do students perceive their dependence on generative AI tools?

## 3 Methods

### 3.1 Sampling and procedure

To comprehensively explore the research issue, this study adopts a mixed-methodology that combines the advantages of quantitative and qualitative methods in the field of education. The quantitative phase is mainly conducted through questionnaires to collect relevant data, and the quantitative results are deeply analyzed to further understand. The qualitative research stage is carried out through in-depth interviews. This study uses the purposeful sampling method, recruiting a total of 266 participants, who are undergraduate students from a public university in Fujian Province, China, and undergraduate students from a university in Guangdong Province, China, all of whom are students in the “Research Methods in Journalism and Communication” course.

The “Research Methods in Journalism and Communication” course is a required course for the journalism major at the school. Writing a literature review is one of the course contents. In this module, students need to learn how to find relevant literature, the components of a literature review, and how to write a qualified literature review. Given the growing integration of AI into higher education, the instructor incorporated AI tools into the delivery of this course content. The teacher introduced some domestic AI tools that can be used directly, such as Douba, DeepSeek, and Kimi, and also introduced the popular foreign tool ChatGPT that can be accessed through third-party mirror websites. We instructed students on how to use AI tools to search for literature, rapidly summarize content, and perform proofreading. Then we assigned relevant course assignments, allowing students to practice AI tools after class. In the submitted assignments, they need to annotate the content generated by AI. In the classroom 2 weeks later, we assigned an on-site assessment as the grade for the midterm assignment. Students need to choose a topic of interest from the 10 relevant topics I provided and complete the writing of the literature review on the spot. To complete the assignment, they need to find relevant literature on the spot, summarize the views of the literature, integrate the materials to expand the discussion, and finally form the final polished version of the literature review paper. Students can choose whether to use AI as an auxiliary tool. The polished paper must follow the Human Factors and Ergonomics Society conference paper template, and students who use AI need to annotate the content generated by AI. The scoring criteria require the paper to have a rigorous structure, strong arguments, thorough understanding, and clear expression. The research was conducted through the distribution of questionnaires and informed consent forms, with participants immediately completing an online questionnaire after finishing the writing of the literature review. To ensure data quality in our teaching, in addition to explaining and demonstrating the advantages and functions of AI software, we also explained the ethical norms and risk prevention measures associated with its use, including the authenticity and reliability of AI-generated data, citation norms, detection methods for AI-generated content, and risk prevention strategies. This approach ensures that students use AI platforms based on a correct understanding, thereby enabling the detection of any dependent behavior issues among them.

A voluntary item was set up after the questionnaire survey to invite respondents willing to participate in follow-up interviews. Subsequently, eight participants were selected for in-depth interviews based on their academic performance levels, thereby ensuring sample heterogeneity. The interviews were conducted using a semi-structured interview guide, with each interview lasting approximately 30 min. After obtaining the informed consent of the respondents, the entire interview process was recorded. The interviews focused on core issues such as students' experience with AI academic writing, motivation for using AI, and views on AI dependence, with typical questions including: “What are the advantages and disadvantages of using AI in academic writing?” “Since you have realized the shortcomings of AI, why are you still using AI?” After the interviews, the recorded materials were fully transcribed into text data. This study used thematic analysis for qualitative data analysis, with two researchers conducting independent open coding and theme extraction, followed by cross-checking of coding results. Discrepancies were discussed item by item, and a consensus was reached to ensure consistency of coding and reliability of analysis.

### 3.2 Measurements

All constructs in the study are derived from mature scales in existing literature and are appropriately adjusted for the context of AI and academic writing. To ensure the accuracy of language and concepts, the original English scales were first translated into Chinese and then back-translated by two bilingual researchers. All items are scored using a five-point Likert scale, ranging from one (strongly disagree) to five (strongly agree). A pre-test with 52 students was conducted to verify the clarity and rationality of each item.

Academic self-efficacy is measured using five items, adapted from Pintrich and Groot (1990) study. A sample item is, “I believe I have the ability to achieve good grades in learning.” These items aim to measure the cognitive situation of the subjects' academic self-efficacy. In this study, this construct shows good internal consistency (α = 0.925). AI literacy is measured using five items adapted from the scale developed by Carolus (Carolus et al., 2023), the modified items aim to measure students' ability to critically and ethically evaluate AI. A sample item is, “I understand the advantages of using AI.” The internal consistency of this construct is relatively high (α = 0.863). Social influence includes three items, mainly referring to the classic questionnaire scale in Venkatesh et al.'s (2003) UTAUT model, the content of the items is appropriately modified according to the AI scenario. A sample item is, “The teacher encourages me to use AI for learning assistance.” After measurement, the reliability of this construct is good (α = 0.853). Academic stress uses five items from the Perceptions of Academic Stress (PAS) scale (Dalia and Adel, 2015), the main items include: “I am unable to catch up if I get behind on the work” and “The competition with my peers for grades is quite intense.” After measurement, this construct shows good internal consistency (α = 0.830). Perceived trust includes three items, adapted from the Human-Computer Trust Scale (Jian et al., 2000), the items are adapted to the generative AI scenario. A sample item is, “I believe AI is honest.” This construct shows good reliability after measurement (α = 0.732). Perceived usefulness is a core construct of the Technology Acceptance Model (TAM), this study uses the Perceived Usefulness Scale (Davis, 1989) and adapts some items according to the use scenario of AI technology, forming four measurement items in the end. A representative item is: “On the whole, I think AI is very helpful to my learning.” Measurement revealed a Cronbach's α of 0.892 for the construct. Generative AI dependence behavior includes seven items, adapted from the Facebook scale (Andreassen et al., 2012) and the LDS dependence scale (Li et al., 2024), revised around the core performance of instrumental dependence and emotional psychological dependence after use. A core item is, “I would feel anxious if I were forbidden to use AI tools” and “I have tried to reduce the use of AI tools in learning, but found it impossible to persist.” After measurement, this construct shows good reliability (α = 0.901).

This study collected demographic information such as participants' gender and educational level. To ensure data quality, we constructed a multi-level questionnaire quality monitoring system to screen out potential invalid feedback. Data that meet any of the following exclusion criteria will not be included in the subsequent analysis: (1) Data missing: if there are omissions in the core questions of the questionnaire, they will be excluded. (2) Regular responses: questionnaires where all items in the scale section are selected with the same option are considered to have insufficient response quality and will be excluded. (3) Short response time: if the time taken to fill out the questionnaire is significantly short, it cannot guarantee the seriousness of the response process, and it will be deleted. (4) Logical consistency check: if there are obvious contradictory option combinations in the questionnaire, they will be excluded after review. This study distributed questionnaires to 266 respondents through the Questionnaire Star platform. Among them, 18 respondents did not participate in answering. Eventually, a total of 248 valid questionnaires were collected. After data review, four incomplete samples were excluded, seven samples with insufficient response time were excluded, and eight straight-line response samples were excluded. After screening, 229 valid samples were retained for analysis.

### 3.3 Data analysis

This study used SPSS 26.0 (IBM Corp., Armonk, NY, USA) and AMOS 26.0 (IBM Corp., Armonk, NY, USA) for quantitative data analysis, combining descriptive and inferential statistical techniques, and tested the influence relationships between key constructs based on the I-PACE model. In the early stages of the study, we first conducted data cleaning and preprocessing to ensure the accuracy and reliability of the data. Through descriptive statistical analysis, we deeply understood the distribution of demographic characteristics and the use of generative AI in the subjects. Subsequently, we tested for common method bias to ensure that the data quality meets the requirements of the analysis. Before conducting confirmatory factor analysis, this study used Mardia's multivariate kurtosis coefficient to test the normality of the data. The results showed that the multivariate kurtosis coefficient was 300.396, the critical ratio was 55.453, far exceeding the recommended threshold of 1.96 (Byrne and Erlbaums, 2009), indicating the presence of significant multivariate non-normality. Considering that maximum likelihood estimation has certain robustness in parameter estimation for non-normal data, but the model chi-square test may be distorted accordingly, this study, while retaining maximum likelihood estimation, corrected the model fitting using 5,000 Bootstrap sample (Bollen and Stine, 1993). We used confirmatory factor analysis (CFA) to evaluate the validity and reliability of the measurement model, ensuring that the definitions of each measurement dimension are clear and internally consistent. Through model fit index evaluation, the robustness of the research framework was verified. We applied structural equation modeling (SEM) to test the hypothetical relationships between variables and systematically analyzed the impact of psychological and behavioral factors on AI dependence. This study aims to reveal the deep mechanisms of excessive use of AI by college students and also provides a theoretical basis and practical guidance for educators to prevent dependence on AI.

## 4 Results

### 4.1 Descriptive statistics

Table 1 shows the demographic results of the participants. 81.2% of the participants were female students, and 18.8% were male students. 30.1% were second-year students, 42.8% of the participants were third-year students, and the rest were fourth-year students. Table 2 presents the usage behavior of the participants toward AI tools. Most students maintained a high frequency of use of AI tools, with a proportion of 48.1% using them frequently, 39.7% using them moderately, 9.6% using them infrequently, and only 2.6% rarely using them. The duration of single use by the respondents was mainly concentrated between “30 min to 1 h” accounting for 57.2% of the total sample, followed by users who used for “less than 30 min,” accounting for 26.6%. Respondents who used for “1–2 h” accounted for 11.4%, while users who used for “more than 2 h” were the least, accounting for 4.8%. Overall, the duration of use was mainly concentrated within 1 h, indicating that most users have a habit of short-term use of AI.

**Table 1:** Demographic characteristics of participants.

Demographic factor	Item	Frequency	Percent (%)
Gender	Male	43	18.8
	Female	186	81.2
Academic year	First-year student	69	30.1
	Second-year student	0	0
	Third-year student	98	42.8
	Fourth-year student	62	27.1

**Table 2:** Participants' AI usage behaviors.

Demographic factor	Item	Frequency	Percent (%)
Frequency of use	High-frequency use: multiple times a day/once a day	110	48.1
	Medium-frequency use: once every 2–3 days/once a week	91	39.7
	Low-frequency use: 2–3 times a month	22	9.6
	Rare use: once a month or less	6	2.6
Duration of use	Less than 30 min	61	26.6
	30 min to 1 h	131	57.2
	1–2 h	26	11.4
	More than 2 h	11	4.8

### 4.2 Common method bias

Since all data were collected from a single source at the same time point, it is necessary to evaluate common method bias. This study adopted two methods to test the common method bias in this batch of cross-sectional data: the Harman's single-factor test and the correlation matrix method. Firstly, the results of the Harman one-factor test showed that the first unrotated factor explained 27.963% of the total variance, which was significantly lower than the 40% critical value suggested by Podsakoff et al. (2003). Secondly, based on the correlation matrix method, the correlation coefficients between the seven constructs ranged from −0.049 to 0.633, all of which were below the critical value of 0.9 (Rasoolimanesh et al., 2021; Yang et al., 2021). Both results indicate that there is no significant common method bias in this single-source data.

### 4.3 Validating the measurement models: confirmatory factor analysis (CFA) model

To ensure that the measurement model meets the testing criteria, the study used AMOS 26.0 software to conduct (CFA). The study presented the CFA results using three dimensions: standardized factor loadings (SFL), composite reliability (CR), and average variance extracted (AVE) (Juan et al., 2022), as shown in Table 3. These data come from a measurement model containing seven constructs, and after CFA validation, all constructs' standardized factor loadings exceed the acceptable threshold of 0.5 (Hair et al., 2020), indicating good internal consistency and convergent validity among the constructs. The composite reliability ranged between 0.728 and 0.925, all exceeding the threshold of 0.7 (Hair et al., 2020). The average variance extracted (AVE) for most constructs was greater than 0.5; the AVE for the TR dimension was 0.476, slightly below the critical standard of 0.5. According to the criteria of Fornell and Larcker (1981), when the composite reliability CR > 0.6, even if AVE is slightly below 0.5, the convergent validity can still be accepted.

**Table 3:** Reliability and validity results.

Construct	Items	SFL	Cronbach's α	CR	AVE
Academic self-efficiency (ASE)	ASE1	0.935	0.925	0.925	0.716
	ASE2	0.952			
	ASE3	0.804			
	ASE4	0.826			
	ASE5	0.687			
AI literacy (AIL)	AIL1	0.852	0.863	0.869	0.575
	AIL2	0.827			
	AIL3	0.808			
	AIL4	0.712			
	AIL5	0.555			
Social influence (SI)	SI1	0.887	0.853	0.856	0.667
	SI2	0.795			
	SI3	0.763			
Academic stress (AS)	AS1	0.758	0.830	0.836	0.508
	AS2	0.713			
	AS3	0.675			
	AS4	0.791			
	AS5	0.612			
Perceived trust (TR)	TR1	0.716	0.732	0.728	0.476
	TR2	0.562			
	TR3	0.776			
Perceived usefulness (PU)	PU1	0.813	0.892	0.894	0.679
	PU2	0.769			
	PU3	0.870			
	PU4	0.842			
AI dependence (AID)	AID1	0.828	0.901	0.897	0.566
	AID2	0.907			
	AID3	0.899			
	AID4	0.778			
	AID5	0.694			
	AID6	0.518			
	AID7	0.539			

The criteria for evaluating discriminant validity require that the square root value of the average variance extracted (expressed as diagonal values) be greater than the correlation coefficients between different constructs (expressed as non-diagonal values) (Fornell and Larcker, 1981). Table 4 shows the results of the correlation matrix, with the square root values of the average variance extracted for all constructs being greater than the correlation coefficients between constructs, providing strong evidence for discriminant validity Confirmatory factor analysis (CFA) indicates that the obtained data is fully applicable to the proposed CFA measurement mode (Akhtar et al., 2024).

**Table 4:** Correlations between variables.

Constructs	ASE	AIL	SI	AS	TR	PU	AID
ASE	0.846						
AIL	0.610	0.758					
SI	0.575	0.620	0.817				
AS	0.383	0.396	0.411	0.713			
TR	0.042	0.154	0.209	0.325	0.690		
PU	0.597	0.609	0.702	0.543	0.157	0.824	
AID	−0.058	−0.006	0.163	0.226	0.343	0.093	0.752

### 4.4 Structural model analysis

To ensure that the current data is consistent with the research model, the model fit is tested. The relevant indicators for evaluating the fit include: (1) absolute fit: chi-square value and approximate root mean square error (RMSEA); (2) incremental fit indices include: standardized fit index (NFI), Tucker–Lewis index (TLI), and comparative fit index (CFI); (3) parsimonious fit index: chi-square/degree of freedom ratio (Ghouri et al., 2024). We conducted fit tests for both the measurement model and the structural model, referring to the modified indices, parameter estimates, and model path coefficients in the Amos output results. Within the principle of not violating empirical rules, we established some error variables with correlation relationships and set residual correlations, formulated a model modification plan. Table 5 showed the statistical results after model modification. The results showed that the Chi-square/degree of freedom ratio, CFI, TLI, and RESEM of the two models all exceed the recommended good fit critical values. Most of the model fit indices indicated that the proposed model fits well.

**Table 5:** Fit statistics for the model.

Model	χ^2^/df	RMSEA	NFI	TLI	CFI
The measurement model	1.841	0.064	0.843	0.904	0.906
The structural model	1.955	0.065	0.854	0.911	0.922
Recommended value	≤ 3.000	≤ 0.08	≥0.9	≥0.9	≥0.9

#### 4.4.1 Testing hypotheses

The research used structural equation models (SEM) to test hypotheses. We used AMOS 26.0 software and standardized estimates, combined with regression weight results to test the research hypotheses. Table 6 showed the standardized regression coefficients of the path analysis in this study, aiming to test the significance of the relationships between variables and evaluate the degree of support for the hypotheses based on the collected data. Table 6 data showed that this research model covers six paths, four of which have statistical significance. Specifically, AS (β = 0.306, *p* < 0.001), AIL (β = −0.196, *p* < 0.05), TR (β = 0.212, *p* < 0.05) are significantly associated with AID, supporting hypotheses **H2, H3, H5**. SI (β = 0.306, *p* < 0.001) is significantly associated with TR, supporting hypothesis H6. ASE has no significant effect on AS (β = 0.050, *p* = 0.500), SI has no significant effect on AID (β = 0.121, *p* = 0.251), rejecting hypotheses **H1** and **H4**.

**Table 6:** Hypotheses test results.

Hypotheses	Path	Estimate	SE	CR	*P*	Result
H1	ASE → AS	0.050	0.078	0.674	0.500	Not supported
H2	AS → AID	0.306	0.086	4.074	^***^	Supported
H3	AIL → AID	−0.0.196	0.142	−1.982	^*^	Supported
H4	SI → AID	0.121	0.153	1.149	0.251	Not supported
H5	TR → AID	0.212	0.116	2.435	^*^	Supported
H6	SI → TR	0.404	0.095	4.606	^***^	Supported

^*^p < 0.05, ^***^p < 0.001.

#### 4.4.2 Mediating effect test

This study explored the mediating role of perceived trust between social influence and generative AI dependence. The study used the corrected bias Bootstrap confidence interval method to test the significance of the mediating effect. If the confidence interval does not include zero, it indicates a significant effect (Preacher and Hayes, 2008). Table 7 showed that there is a significant mediating effect of perceived trust between social influence and dependence behavior. The effect value of the indirect effect is 0.124, and the 95% confidence interval ranged from 0.015 to 0.343, excluding zero. The direct effect coefficient of social influence on AI dependence is 0.176, and the 95% confidence interval ranged from −0.193 to 0.492, including zero. The total effect value is 0.300, and the 95% confidence interval ranged from −0.038 to 0.635, including zero, supporting hypothesis H7. The study results indicated that there is no significant direct effect of social influence on AI dependence, social influence indirectly affected AI dependence through perceived trust.

**Table 7:** Mediation effect testing.

Effect	Path	Estimate	Bias-corrected 95% CI		*P*	Result
			Lower	Upper		
Indirect effect	SI → TR → AID	0.124	0.015	0.343	0.023	Supported
Direct effect	SI → AID	0.176	−0.193	0.492	0.303	Not supported
Total effect	SI → AID	0.300	−0.038	0.635	0.078	Not supported

#### 4.4.3 Testing the moderating effect

This study considered the moderating effect of perceived usefulness on the relationship between academic stress (dependent variable) and academic self-efficacy (independent variable). A model was established using the statistical software SPSS 26.0 (IBM Corp., Armonk, NY, USA) to test this moderating effect. ASE, AS, and PU were all standardized before analysis. An interaction term (ASE^*^PU) was generated to capture the interaction between the independent variable ASE and the moderating variable PU. The results shown in Table 8 indicate that the regression coefficient of PU on AS is 0.2335, with a *p*-value of 0.0184. A *p*-value less than 0.05 indicates that PU has a statistically significant effect on AS (Li et al., 2024), confirming that PU has a positive and statistically significant effect on AS. The current analysis shows that at the significance level *p* < 0.05, the regression coefficient of the interaction term ASE^*^PU on AS is −0.1732, with a *p*-value of 0.0317. This result indicates a significant moderating effect, suggesting that the relationship between ASE and AS is negatively moderated by PU.

**Table 8:** Moderating effects testing.

Constructs	Effect	SE	*t*-value	*P*	LLCI	ULCI
Constant	0.046	0.0502	0.9158	0.3608	−0.053	0.145
ASE	−0.0892	0.0745	−1.1965	0.2328	−0.236	0.0577
PU	0.2335	0.0983	2.3743	0.0184	0.0397	0.4272
ASE^*^PU	−0.1733	0.0802	−2.1616	0.0317	−0.3313	−0.0153

### 4.5 Qualitative results

In addition to quantitative results, this study obtained some data on college students' feedback, evaluations, and cognitive awareness of dependence behavior regarding the use of generative AI through the setting of open-ended questions and conducting interviews. We imported the text results of the open-ended questions into the Weiciyun online platform for word frequency analysis. In the preprocessing phase, irrelevant symbols and unanswered text were removed. The results of the word frequency analysis show that students believe the biggest flaw of generative AI is that AI can forge data and it is difficult to judge the authenticity of the generated content. Among them, the common views mentioned by students include: obvious AI traces, privacy leakage, and inability to understand instructions.

We adopted the thematic analysis method to analyze the data, and after coding and categorization, we obtained four themes under two classification dimensions. Under the category of generative AI usage motivation, two themes were revealed: efficiency and peer influence. Students consistently believed that the efficiency of AI is a major advantage. Using AI can help them cope with heavy homework, tight deadlines, and continuous pressure, and students are turning to AI to save time and improve efficiency. Some interviewees mentioned: “AI can help me quickly summarize the content and conclusions of literature, and my efficiency in completing assignments doubles.” Another interviewee described her experience with AI: “When I am stuck in writing, I use AI to quickly get inspiration, which is really practical.” Another interviewee expressed: “Just by giving instructions, AI can provide answers, which is simply stunning, especially when the deadline is approaching.” Additionally, some interviewees used AI to revise their assignments: “When I don't know what to add, AI automatically expands and rewrites my paper.” We found that some students use AI due to peer influence. “My classmates are all using AI, and if I don't use it, our academic gap may be widened.” “Some students use DeepSeek to deal with papers, and the AI-generated articles have a rigorous structure and smooth content, and they often get high scores, while my own papers get lower scores.”

Under the category of perceived Generative AI dependence, we identified two key themes: confidence and rule ambiguity. Students generally believe that they can master AI and clearly define its boundaries of use, and they believe they will not misuse AI. Some interviewees mentioned that they only use AI in a few theoretical courses: “I only use AI in theoretical courses, those technical or operational courses cannot use AI, so I will not develop a dependence on AI.” Although many students are confident in their ability to skillfully use AI tools, they believe their strategies are clever and will not be completely dependent on the results of AI output. They are good at adjusting the content generated by AI to avoid being noticed by plagiarism detection systems, professors, and classmates. One student shared his strategy: “I will use various tools such as ChatGPT, DeepSeek, and Douba, mix the output results, adjust the order, and optimize the final result.” In addition to confidence, students also widely use various loop mechanisms. Set relevant mechanisms to ensure that students will not be penalized even if they overuse AI. Some students said: “Although teachers require us to use AI responsibly, there is no clear definition of ‘what extent of use can be considered as non-compliant' at present.” One interviewee pointed out: “Although the school claims to monitor our usage, teachers have not yet adopted AI plagiarism detection tools to check assignments.” Some students also mentioned: “I will use different AI tools to reorganize the content to make it sound more like my original writing; while teachers cannot distinguish my original work from the content generated by AI with the naked eye.” Our results show that the ambiguity at the institutional level, the unclear policies, and the inconsistency between policies have blurred students' understanding of the reasonable use guidelines for generative AI.

## 5 Discussion

### 5.1 Factors influencing college students' dependence on generative AI (research question 1)

This section discusses research question 1 (RQ1): what factors are related to students' dependence on generative AI during the writing process? This study, combining the interpretation of existing literature, fully reveals the psychological and behavioral driving factors behind excessive dependence on generative AI in the educational context.

Analysis confirms that academic stress is the strongest predictive factor for the generation of AI dependence in college students' academic writing courses, which is consistent with the research of Liu et al. (2026) and Zhang et al. (2024). The stronger the academic stress, the more likely users are to seek external support to alleviate stress, with AI tools capable of quickly generating content and providing learners with quick and convenient consulting services and other academic assistance. The perceived value of AI tools by individuals with high academic stress enhances the possibility of functional use evolving into problematic behaviors, as high-stress individuals may find it increasingly difficult to separate academic tasks from AI assistance.

Perceived trust is also a key factor in predicting college students' dependence on generative AI. Although we have consciously revealed the errors and risks of AI in our experiments, we find that these risks do not necessarily lead people to hold negative views or stop using related software, which is consistent with the views of Kaya et al. (2022). Generative AI sometimes fails to handle complex logical reasoning, producing seemingly reasonable but actually false “facts.” This “false fact” is difficult to distinguish from the truth due to its concealment and realistic form, easily forming a “hallucination” effect on users. Although college students have a certain level of risk and ethical awareness, they cannot screen the reliability of AI content and the integrity of resources, leading to excessive cognitive and emotional trust and forming an AI dependence syndrome. This also verifies the view of Lucas that people often believe they have enough knowledge to understand AI, thus being able to identify its potential risks and avoid dependence (Lucas et al., 2024).

Research confirms that AI literacy has a negative predictive effect on college students' dependence on generative AI. Learners with lower AI literacy are more prone to psychological dependence in academic tasks, which is consistent with the conclusions of the studies by Zhang et al. (2024) and Ling (2025). AI literacy not only cultivates technical understanding but also enhances psychological resilience, helping users alleviate fear, avoid blind trust, and interact with AI technology in a more autonomous and informed manner. Effective AI literacy can help people use AI responsibly and maintain mental health. This study confirms that learners with lower AI literacy are more likely to develop psychological dependence in academic tasks.

It is noteworthy that we did not find a significant correlation between academic self-efficacy and academic stress, which is in contrast to the previous research (Odaci, 2011; Parmaksiz, 2022). This finding indicates that low academic performance does not necessarily lead to an increase in academic stress, thereby triggering the emergence of AI problem behaviors. However, this study finds an indirect relationship between academic self-efficacy and academic stress, which is moderated by perceived usefulness. In high perceived usefulness situations, the negative impact of academic self-efficacy on academic stress is significantly enhanced. This may mean that when students highly recognize the value of using AI tools, students with high self-efficacy are more able to effectively utilize these tools, thereby better managing academic requirements and significantly reducing stress experiences. Conversely, when students' perception of the utility of generative AI is at a moderate or low level, there is no significant correlation between academic self-efficacy and academic stress. This indicates that the buffering effect of academic self-efficacy on academic stress does not necessarily occur and depends on individuals' positive perception of the “utility” of AI tools. High perceived utility may be a necessary condition to activate the “stress buffering effect” of self-efficacy. In other words, even if students have a high level of academic self-efficacy, if they fail to perceive the practical value of AI tools, this sense of efficacy is difficult to transform into actual stress relief effects.

The direct path of social influence on AI dependence is not significant, which contradicts the view in the UTAUT theory that social influence affects technology adoption and use. The qualitative data of this study also verifies the positive effect of peer and key person influence on AI use. This suggests that social influence is not a direct influence on students' dependence behavior. The words and actions of peers or key individuals may influence technology adoption, but this does not necessarily lead to dependence behavior. The mediation test results reveal that social influence needs to rely on the transmission of perceived trust to act on dependence behavior. This indicates that the opinions and behaviors of peers and important others will not directly prompt individuals to form AI dependence; only when it is internalized as a stable perception of trust, it is more likely to produce persistent and habitual dependence behavior. This also explains why the qualitative interviews can observe the driving effect of social influence on technology use behavior, but the direct path of social influence on AI dependence is not significant in the quantitative results: social influence is more likely to initiate general technology adoption, and it takes individual cognitive beliefs as a key mediating bridge to go from routine use to behavioral dependence. This conclusion further indicates that when explaining AI dependence, the mediating transmission mechanism of individual subjective perception beliefs cannot be ignored.

The research results generally support the applicability of the I-PACE framework in AI usage scenarios. The framework was initially used to explain the barriers to the use of hedonic applications such as social media and games, and this study extends it to the use of AI tools in academic writing. Specifically, this study introduces the variable of AI literacy in the individual dimension of I-PACE, which is not a pathological trait but a malleable ability factor, enriching the framework's understanding of individual preconditions. Secondly, this study identifies a path from personal traits through emotional and cognitive processing to behavioral outcomes. Specifically, the path from social influence to perceived trust to AI dependence further confirms the mechanism in the I-PACE framework where individual traits drive behavioral outcomes through emotional and cognitive processing.

### 5.2 What are the motivations of students to use generative AI tools in academic writing, and how do students perceive their dependence on generative AI tools? (research question 2)

Qualitative research findings show that despite many concerns about AI, students with negative attitudes toward AI have not stopped using it, which is consistent with the research results of (Kaya et al. 2022). Efficiency priority and peer influence become important drivers for students to use AI, while academic pressure further prompts students to weigh risks and benefits, leading them to prioritize task efficiency and weaken their vigilance toward the risks of AI use under the influence of multiple factors. They continue to use AI technology in academic writing. At the perception level of students' dependence on generative AI, the research finds that students' cognition of AI dependence is still relatively superficial. When mentioning risks, most of them express the limitations and risk points of AI tools, but are not aware of the association between AI and cognitive offloading, as few people mention that excessive dependence on AI may weaken thinking and self-learning abilities, etc. They believe that using AI only when needed does not constitute excessive dependence. We also found that students generally have a strategic confidence in evading AI detection, using AI strategically through a combination of multiple tools and text rewriting, and they do not consider this as unethical or dependent behavior. This finding confirms the view of Seran et al.: the cognitive dissonance of dependence relationships prompts individuals to construct rationalizing attributions, thereby providing a legitimate reason for academic misconduct. However, institutions and educators have not clearly defined the boundaries of AI use, and the vague system exacerbates this issue, which requires more attention in subsequent education.

## 6 Implications and limitations

### 6.1 Theoretical significance

This study integrates key variables from various theories into the I-PACE model, focusing on academic writing within the curriculum constraint, and provides a new theoretical framework for understanding the motivations that influence the generative AI dependence behavior of the college student group. The I-PACE model has been widely applied in scenarios such as online games, smartphones, and social media, but its application in the educational field is still very limited. This study fills the gap in the research on generative AI dependence in college students' writing tasks by applying the I-PACE model, focusing on the psychological mechanisms and influencing factors that affect their dependence.

Perceiving usefulness and academic stress as key motivators at the emotional and cognitive levels in the I-PACE model adds a new dimension to the discussion on AI-dependent behavior, revealing how emotions and cognition drive students from tool use to deep dependence. The study finds that academic stress is the strongest influencing factor for AI-dependent behavior, while perceived usefulness has a regulatory constraint effect on academic stress for different self-efficacy groups. This finding enriches the interpretation of the relationship between academic self-efficacy and academic stress.

This qualitative study reveals that the excessive use of AI in writing by students is also related to the complex interaction of utilitarian pragmatism, institutional norms, and loopholes. In addition to psychological factors, attention should also be paid to the influence of the above factors. The research expands theoretical cognition, prompting future research to simultaneously focus on the interaction between internal psychological factors and external mechanisms, providing a theoretical guide to avoid excessive dependence on AI.

### 6.2 Practical significance

This study provides practical guidance for educators, institutions, and policymakers. At the level of educators, emphasis should be placed on addressing student behavioral deviations caused by academic stress, which requires educators to genuinely reduce students' academic burden and create a learning environment that prioritizes learning and complements task completion. It is important to cultivate college students' AI literacy and risk perception awareness, as there is already empirical evidence that both AI literacy and risk awareness are key paths for predicting dependence on AI. Students need to maintain a critical understanding of AI to ensure the distinction between strategic use and habitual use. Educators should integrate AI into the curriculum from an ethical perspective, cultivating students' critical thinking and responsible use awareness. Educators need to systematically integrate risk awareness training into the curriculum, fostering students' predictive evaluation abilities for potential technological risks.

At the level of educational institutions, clear guidelines and supervision mechanisms must be established to ensure that AI is used as an auxiliary tool rather than a substitute for professional skills. The evasive strategies implemented by students in this study, through the integration of various AI tools and rewriting techniques, indicate that the existing detection methods are clearly insufficient. The education department should consider collaborating with AI developers to strengthen detection while designing reflective prompts to promote conscious and proactive participation (Hu et al., 2023; Zhong et al., 2024). Educational institutions must shift from passive response to proactive prevention, addressing the root problems of AI misuse through systematic ethical education.

At the policy level, the education authorities should further clarify the policy requirements for the integration of AI into the curriculum and propose operational guidelines. The policy document should explicitly stipulate: in the teaching assisted by AI, the manual supervision link must be retained, and teachers should review and evaluate the process and results of students using AI. At the same time, policymakers should also list clear scenarios where AI is not applicable. For example, in tasks involving original expression, critical thinking training, experimental data collection, manual skill operation, and group collaboration reflection, the use of AI should be restricted or prohibited.

### 6.3 Limitations and future directions

This study has several limitations that may affect the interpretation of the results. This study has several limitations that may affect the interpretation of the results. Firstly, this study was conducted under the same academic curriculum system of two universities in China. The sample size of this study is relatively limited. This context-specific sampling method limits the generalizability of the research findings. The differences among universities in different regions in terms of institutional policies, curriculum characteristics, infrastructure conditions, and attitudes toward AI may have an impact on student behavior that is not covered by this study. Future research should include samples from multiple disciplines and conduct research in more universities and regions. Moreover, there is a significant imbalance in the gender composition of the sample, with a higher proportion of females, which limits the external validity of the research conclusions when generalized to the male population. This gender distribution characteristic aligns with the actual structure of students in the field of journalism and communication in China, but due to the limited size of the male sample, it is difficult to conduct reliable multi-group structural equation model analysis and test for gender differences in the model paths. Future research can adopt stratified sampling to include samples with a more balanced gender structure and further investigate the gender differences in the formation mechanism of AI dependence. This will help to verify the applicability of the theoretical model proposed in this study to a wider population, thereby promoting an understanding of problem behaviors in generative AI.

Secondly, all participants come from the same course and have received unified AI skill training before data collection. Although this can enhance students' familiarity and usage tendency toward AI tools, it also constitutes a potential threat to internal validity: the AI dependence levels observed in this study may be influenced by the exposure to course teaching, making it difficult to distinguish between instrumental use in task situations and long-term spontaneously formed habitual dependence. Future research can adopt multi-wave longitudinal tracking or group comparative experiments to avoid the confusion and interference caused by course teaching, distinguish between task-based temporary use and habitual AI dependence, accurately explore the formation mechanism of AI dependence, and improve the internal and external validity of the research.

This study uses cross-sectional self-reported questionnaire data, a measurement method that cannot infer the causal relationship between variables. Although the research data support certain theoretical framework paths, the associations between them should be interpreted as correlations consistent with the proposed model, rather than causal relationships. At the same time, the measurement tool is single, and the study mainly relies on the self-assessment scales of the subjects, without combining AI background logs, classroom behavior observations, and other objective data for cross-validation. Single self-reported measurements are prone to bias and cannot accurately restore the students' real AI dependence behavior. Future research can adopt longitudinal tracking designs or set up control groups, combining system log data, screen recording, or observation techniques to analyze behavioral data, in order to more accurately reveal the complex mechanisms behind them.

This study's qualitative data is subject to certain validity constraints due to the limited size of the qualitative sample, the restricted representativeness of respondents' views, and the difficulty in directly generalizing to a larger student population. Although this study controlled for coding subjectivity through multiple coders' cross-checking and respondent feedback verification, it was still unable to completely eliminate the impact of researcher judgment bias. Subsequent research can expand the qualitative sample and adopt more systematic qualitative validation methods to further enhance the effectiveness of qualitative data. The dependence of generative AI in academic writing contexts is an emerging research issue, with existing theories and empirical accumulations insufficient to support all path hypotheses under the I-PACE framework. Constrained by both the theoretical derivation foundation and sample size, this study only selected core variables with empirical support to construct the model and did not include all potential influencing factors under this framework. This model simplification may lead to an incomplete understanding of the formation mechanism of generative AI dependence. Future research can include more variables, test more complex path relationships, and further improve the application of the I-PACE framework in the academic use of AI.

## 7 Conclusion

This study explores the use of AI tools by college students in academic writing tasks and the psychological and behavioral mechanisms behind their dependence. The research reveals how factors such as academic self-efficacy, AI literacy, perceived trust, academic stress, and perceived usefulness directly or indirectly affect the emergence of dependence behaviors. The core insight of this study lies in revealing the transmission mechanism from social influence to perceived trust, and it emphasizes that academic stress and AI literacy are significant influencing factors. In educational and professional environments, personal traits and task-related involvement can develop dependence through the reinforcing effects of emotions and cognition, thereby revealing the mechanism of technology dependence.

Research also finds that dependence behaviors in the field of AI encompass profound emotional, cognitive, social, and cultural dimensions, such as complex interactions between personal confidence, system vulnerabilities, and policy ambiguity, which is not simply a psychological level issue. Therefore, focusing solely on psychological level factors is insufficient to address the inappropriate behaviors of AI. This research not only fills the gap in current literature on AI dependence research but also focuses on multiple dimensions, providing a new perspective for digital dependence research in the field of AI. By analyzing these correlations, the research provides timely insights for educators, school administrators, and policymakers, aiming to responsibly integrate AI into teaching.

## Data Availability

The original contributions presented in the study are included in the article/Supplementary material. Further inquiries can be directed to the corresponding author/s.
