# Supplementary material for: Are students dependent on AI in writing courses? Analyzing factors influencing dependence on generative AI through the I-PACE model
Source: Front Psychol. 2026 Sep 11;17:1905037. doi: 10.3389/fpsyg.2026.1905037 (PMC13612266; doi:10.3389/fpsyg.2026.1905037)
Supplement: Supplementary file 1 [file Data_Sheet_1.zip › my supplementary file/Appendix A.docx]

**Appendix A**

**A Survey on College Students' Perceptions of AI-Assisted Learning**

| **Academic Self-Efficacy** | I believe I have the ability to achieve good grades in learning. |
| --- | --- |
|  | I think I am capable of solving problems I encounter in my studies. |
|  | Compared with other classmates, I have relatively strong learning ability. |
|  | I believe I can well understand knowledge from textbooks and the content taught by teachers. |
|  | Whether my academic performance is good or poor, I never doubt my learning ability. |
| **Academic Stress** | The course workload is heavy, and I have too many assignments. |
|  | I am unable to catch up if I get behind on the work. |
|  | The competition with my peers for grades is quite intense. |
|  | The assignments are quite challenging. |
|  | I am worried about my unsatisfactory academic performance. |
| **AI Literacy** | I understand the advantages of using AI. |
|  | I am aware of the limitations of AI application. |
|  | When deciding whether to adopt AI-generated data, I consider ethical issues (such as data authenticity and plagiarism risks). |
|  | I verify the authenticity of data provided by AI. |
|  | I can tell apart AI-generated content and non-AI-generated content. |
| **Social Influence** | News, online media and schools encourage me to use AI. |
|  | The teacher encourages me to use AI for learning assistance. |
|  | My peers often use AI to assist their studies. |
| **Perceived Trust** | I believe AI is honest. |
|  | I trust that AI will not expose personal data or compromise privacy. |
|  | I believe that, given appropriate input prompts, AI can reliably generate the outcomes I expect. |
| **Perceived Usefulness** | I think AI helps me solve problems more effectively. |
|  | Interacting with AI offers more support and guidance than traditional learning methods. |
|  | Using AI for learning can boost my learning efficiency. |
|  | On the whole, I think AI is very helpful to my learning. |
| **AI Dependence** | I find it hard to focus on tasks without assistance from AI tools. |
|  | I would feel restless if the use of AI tools were prohibited. |
|  | I would feel anxious if I were forbidden to use AI tools. |
|  | I am unwilling to begin my work without AI tools. |
|  | I have tried to reduce the use of AI tools in learning, but found it impossible to persist |
|  | Frequent use of AI tools negatively affects my study and daily life (I rely less on independent thinking, and my critical thinking is limited). |
|  | When facing difficulties, I immediately seek help from AI. |
