# Supplementary material for: Are students dependent on AI in writing courses? Analyzing factors influencing dependence on generative AI through the I-PACE model
Source: Front Psychol. 2026 Sep 11;17:1905037. doi: 10.3389/fpsyg.2026.1905037 (PMC13612266; doi:10.3389/fpsyg.2026.1905037)
Supplement: Supplementary file 1 [file Data_Sheet_1.zip › my supplementary file/SEM.AmosOutput]

SEM.amw


# C:\Users\PTXY\Desktop\补充材料\SEM.amw

## Analysis Summary

## Date and Time

Date: 2026年8月20日

Time: 14:12:51

## Title

Sem: 2026年8月20日 14:12

## Groups

## Group number 1 (Group number 1)

## Notes for Group (Group number 1)

The model is recursive.

Sample size = 229

## Variable Summary (Group number 1)

## Your model contains the following variables (Group number 1)

Observed, endogenous variables

ASE5

ASE4

ASE3

ASE2

ASE1

AIL4

AIL3

AIL2

AIL1

SI3

SI2

SI1

TR3

TR2

TR1

AS1

AS2

AS3

AS4

AID1

AID2

AID3

AID4

AID5

AS5

AID6

AID7

AIL5

Unobserved, endogenous variables

TR

ASS

AID

Unobserved, exogenous variables

ASE

e1

e2

e3

e4

e5

AIL

e6

e7

e8

e9

SI

e10

e11

e12

e13

e14

e15

e16

e17

e18

e19

e20

e21

e22

e23

e24

e25

e26

e27

e28

e29

e30

e31

## Variable counts (Group number 1)

|  |  |
| --- | --- |
| Number of variables in your model: | 65 |
| Number of observed variables: | 28 |
| Number of unobserved variables: | 37 |
| Number of exogenous variables: | 34 |
| Number of endogenous variables: | 31 |

## Parameter Summary (Group number 1)

|  | Weights | Covariances | Variances | Means | Intercepts | Total |
| --- | --- | --- | --- | --- | --- | --- |
| Fixed | 37 | 0 | 0 | 34 | 3 | 74 |
| Labeled | 3 | 0 | 0 | 0 | 0 | 3 |
| Unlabeled | 25 | 13 | 34 | 0 | 28 | 100 |
| Total | 65 | 13 | 34 | 34 | 31 | 177 |

## Assessment of normality (Group number 1)

| Variable | min | max | skew | c.r. | kurtosis | c.r. |
| --- | --- | --- | --- | --- | --- | --- |
| AIL5 | 1.000 | 5.000 | -.348 | -2.153 | .049 | .151 |
| AID7 | 1.000 | 5.000 | -.294 | -1.818 | -.455 | -1.405 |
| AID6 | 1.000 | 5.000 | -.245 | -1.511 | -.412 | -1.273 |
| AS5 | 1.000 | 5.000 | -.487 | -3.010 | -.284 | -.878 |
| AID5 | 1.000 | 5.000 | -.193 | -1.192 | -.578 | -1.786 |
| AID4 | 1.000 | 5.000 | .192 | 1.185 | -.415 | -1.282 |
| AID3 | 1.000 | 5.000 | -.116 | -.716 | -.696 | -2.150 |
| AID2 | 1.000 | 5.000 | -.181 | -1.117 | -.762 | -2.353 |
| AID1 | 1.000 | 5.000 | .017 | .104 | -.428 | -1.321 |
| AS4 | 1.000 | 5.000 | -.206 | -1.273 | -.014 | -.043 |
| AS3 | 1.000 | 5.000 | -.043 | -.264 | -.120 | -.369 |
| AS2 | 1.000 | 5.000 | .049 | .303 | -.593 | -1.833 |
| AS1 | 1.000 | 5.000 | -.316 | -1.952 | .218 | .673 |
| TR1 | 1.000 | 5.000 | -.192 | -1.188 | .109 | .337 |
| TR2 | 1.000 | 5.000 | .107 | .662 | -.012 | -.036 |
| TR3 | 1.000 | 5.000 | -.341 | -2.106 | .127 | .393 |
| SI1 | 2.000 | 5.000 | -.327 | -2.022 | .102 | .315 |
| SI2 | 2.000 | 5.000 | -.361 | -2.232 | .051 | .156 |
| SI3 | 2.000 | 5.000 | -.485 | -2.997 | .147 | .455 |
| AIL1 | 1.000 | 5.000 | -1.151 | -7.112 | 2.703 | 8.350 |
| AIL2 | 1.000 | 5.000 | -.770 | -4.760 | 1.733 | 5.354 |
| AIL3 | 1.000 | 5.000 | -.685 | -4.234 | .737 | 2.277 |
| AIL4 | 1.000 | 5.000 | -.671 | -4.147 | .635 | 1.960 |
| ASE1 | 1.000 | 5.000 | -.880 | -5.438 | 1.579 | 4.877 |
| ASE2 | 1.000 | 5.000 | -.992 | -6.127 | 2.063 | 6.373 |
| ASE3 | 1.000 | 5.000 | -.422 | -2.605 | .413 | 1.276 |
| ASE4 | 1.000 | 5.000 | -.770 | -4.758 | 1.124 | 3.471 |
| ASE5 | 1.000 | 5.000 | -.646 | -3.991 | .342 | 1.057 |
| Multivariate |  |  |  |  | 300.396 | 55.453 |

## Observations farthest from the centroid (Mahalanobis distance) (Group number 1)

| Observation number | Mahalanobis d-squared | p1 | p2 |
| --- | --- | --- | --- |
| 145 | 113.879 | .000 | .000 |
| 3 | 99.669 | .000 | .000 |
| 46 | 99.393 | .000 | .000 |
| 87 | 91.575 | .000 | .000 |
| 123 | 84.081 | .000 | .000 |
| 174 | 81.180 | .000 | .000 |
| 178 | 69.782 | .000 | .000 |
| 11 | 69.544 | .000 | .000 |
| 156 | 66.177 | .000 | .000 |
| 8 | 64.109 | .000 | .000 |
| 219 | 60.939 | .000 | .000 |
| 170 | 60.767 | .000 | .000 |
| 55 | 59.848 | .000 | .000 |
| 153 | 59.039 | .001 | .000 |
| 213 | 58.382 | .001 | .000 |
| 131 | 58.281 | .001 | .000 |
| 136 | 57.204 | .001 | .000 |
| 111 | 55.940 | .001 | .000 |
| 15 | 54.822 | .002 | .000 |
| 216 | 54.083 | .002 | .000 |
| 9 | 54.027 | .002 | .000 |
| 83 | 53.865 | .002 | .000 |
| 71 | 52.831 | .003 | .000 |
| 2 | 51.808 | .004 | .000 |
| 37 | 51.725 | .004 | .000 |
| 102 | 51.449 | .004 | .000 |
| 38 | 51.431 | .004 | .000 |
| 39 | 50.768 | .005 | .000 |
| 135 | 50.159 | .006 | .000 |
| 187 | 49.419 | .008 | .000 |
| 66 | 48.846 | .009 | .000 |
| 16 | 48.523 | .009 | .000 |
| 215 | 48.354 | .010 | .000 |
| 183 | 47.101 | .013 | .000 |
| 227 | 46.535 | .015 | .000 |
| 31 | 45.734 | .019 | .000 |
| 91 | 44.320 | .026 | .000 |
| 151 | 43.367 | .032 | .000 |
| 100 | 43.177 | .033 | .000 |
| 149 | 41.851 | .045 | .000 |
| 164 | 41.695 | .046 | .000 |
| 225 | 41.398 | .049 | .000 |
| 222 | 40.355 | .061 | .000 |
| 195 | 38.966 | .081 | .000 |
| 184 | 38.931 | .082 | .000 |
| 175 | 38.732 | .085 | .000 |
| 155 | 38.684 | .086 | .000 |
| 92 | 38.576 | .088 | .000 |
| 201 | 38.139 | .096 | .000 |
| 205 | 37.495 | .108 | .000 |
| 186 | 37.487 | .109 | .000 |
| 167 | 37.016 | .119 | .000 |
| 56 | 36.971 | .120 | .000 |
| 57 | 36.771 | .124 | .000 |
| 144 | 36.731 | .125 | .000 |
| 72 | 36.633 | .127 | .000 |
| 125 | 36.591 | .128 | .000 |
| 95 | 36.393 | .133 | .000 |
| 53 | 36.328 | .134 | .000 |
| 220 | 36.283 | .136 | .000 |
| 194 | 35.961 | .144 | .000 |
| 130 | 35.886 | .146 | .000 |
| 122 | 35.351 | .160 | .000 |
| 5 | 35.055 | .168 | .000 |
| 171 | 34.868 | .174 | .000 |
| 112 | 34.390 | .188 | .000 |
| 65 | 33.786 | .208 | .002 |
| 70 | 33.572 | .215 | .002 |
| 118 | 33.015 | .235 | .013 |
| 148 | 32.816 | .243 | .017 |
| 190 | 32.339 | .261 | .055 |
| 36 | 32.215 | .266 | .058 |
| 116 | 32.202 | .266 | .045 |
| 211 | 31.812 | .282 | .098 |
| 161 | 31.794 | .283 | .079 |
| 76 | 31.632 | .290 | .092 |
| 137 | 31.197 | .308 | .200 |
| 154 | 30.873 | .323 | .304 |
| 1 | 30.502 | .340 | .457 |
| 49 | 30.441 | .342 | .437 |
| 104 | 30.200 | .354 | .525 |
| 176 | 29.906 | .368 | .643 |
| 4 | 29.827 | .372 | .636 |
| 152 | 29.800 | .373 | .599 |
| 197 | 29.625 | .381 | .649 |
| 173 | 29.543 | .385 | .644 |
| 30 | 29.414 | .392 | .667 |
| 59 | 29.337 | .396 | .660 |
| 54 | 29.221 | .401 | .676 |
| 52 | 28.895 | .418 | .796 |
| 33 | 28.782 | .424 | .808 |
| 199 | 28.749 | .425 | .785 |
| 81 | 28.554 | .435 | .832 |
| 115 | 28.498 | .438 | .820 |
| 107 | 28.207 | .454 | .893 |
| 88 | 27.657 | .483 | .977 |
| 97 | 27.500 | .491 | .983 |
| 191 | 27.457 | .494 | .980 |
| 94 | 27.227 | .506 | .989 |
| 113 | 27.005 | .518 | .994 |

## Models

## Default model (Default model)

## Notes for Model (Default model)

## Computation of degrees of freedom (Default model)

|  |  |
| --- | --- |
| Number of distinct sample moments: | 434 |
| Number of distinct parameters to be estimated: | 103 |
| Degrees of freedom (434 - 103): | 331 |

## Result (Default model)

Minimum was achieved

Chi-square = 647.135

Degrees of freedom = 331

Probability level = .000

## Group number 1 (Group number 1 - Default model)

## Estimates (Group number 1 - Default model)

## Scalar Estimates (Group number 1 - Default model)

## Maximum Likelihood Estimates

## Regression Weights: (Group number 1 - Default model)

|  |  |  | Estimate | S.E. | C.R. | P | Label |
| --- | --- | --- | --- | --- | --- | --- | --- |
| ASS | <--- | ASE | .053 | .078 | .674 | .500 | par\_22 |
| TR | <--- | SI | .440 | .095 | 4.606 | \*\*\* | a |
| AID | <--- | ASS | .351 | .086 | 4.074 | \*\*\* | par\_23 |
| AID | <--- | AIL | -.281 | .142 | -1.982 | .048 | par\_24 |
| AID | <--- | SI | .176 | .153 | 1.149 | .251 | c |
| AID | <--- | TR | .282 | .116 | 2.435 | .015 | b |
| ASE5 | <--- | ASE | 1.000 |  |  |  |  |
| ASE4 | <--- | ASE | 1.042 | .085 | 12.269 | \*\*\* | par\_4 |
| ASE3 | <--- | ASE | 1.086 | .087 | 12.534 | \*\*\* | par\_5 |
| ASE2 | <--- | ASE | 1.206 | .099 | 12.209 | \*\*\* | par\_6 |
| ASE1 | <--- | ASE | 1.229 | .102 | 12.028 | \*\*\* | par\_7 |
| AIL4 | <--- | AIL | 1.000 |  |  |  |  |
| AIL3 | <--- | AIL | 1.108 | .089 | 12.427 | \*\*\* | par\_8 |
| AIL2 | <--- | AIL | 1.121 | .112 | 10.000 | \*\*\* | par\_9 |
| AIL1 | <--- | AIL | 1.248 | .121 | 10.271 | \*\*\* | par\_10 |
| SI3 | <--- | SI | 1.000 |  |  |  |  |
| SI2 | <--- | SI | 1.101 | .095 | 11.549 | \*\*\* | par\_11 |
| SI1 | <--- | SI | 1.210 | .097 | 12.416 | \*\*\* | par\_12 |
| TR3 | <--- | TR | 1.000 |  |  |  |  |
| TR2 | <--- | TR | 1.022 | .139 | 7.340 | \*\*\* | par\_13 |
| TR1 | <--- | TR | 1.238 | .161 | 7.692 | \*\*\* | par\_14 |
| AS1 | <--- | ASS | 1.000 |  |  |  |  |
| AS2 | <--- | ASS | 1.038 | .102 | 10.158 | \*\*\* | par\_15 |
| AS3 | <--- | ASS | .947 | .099 | 9.567 | \*\*\* | par\_16 |
| AS4 | <--- | ASS | 1.003 | .090 | 11.125 | \*\*\* | par\_17 |
| AID1 | <--- | AID | 1.000 |  |  |  |  |
| AID2 | <--- | AID | 1.025 | .078 | 13.100 | \*\*\* | par\_18 |
| AID3 | <--- | AID | 1.000 | .078 | 12.853 | \*\*\* | par\_19 |
| AID4 | <--- | AID | 1.011 | .065 | 15.534 | \*\*\* | par\_20 |
| AID5 | <--- | AID | .830 | .074 | 11.250 | \*\*\* | par\_21 |
| AS5 | <--- | ASS | .966 | .111 | 8.716 | \*\*\* | par\_25 |
| AID6 | <--- | AID | .586 | .078 | 7.480 | \*\*\* | par\_29 |
| AID7 | <--- | AID | .597 | .077 | 7.709 | \*\*\* | par\_30 |
| AIL5 | <--- | AIL | .815 | .104 | 7.851 | \*\*\* | par\_31 |

## Standardized Regression Weights: (Group number 1 - Default model)

|  |  |  | Estimate |
| --- | --- | --- | --- |
| ASS | <--- | ASE | .050 |
| TR | <--- | SI | .404 |
| AID | <--- | ASS | .306 |
| AID | <--- | AIL | -.196 |
| AID | <--- | SI | .121 |
| AID | <--- | TR | .212 |
| ASE5 | <--- | ASE | .651 |
| ASE4 | <--- | ASE | .801 |
| ASE3 | <--- | ASE | .772 |
| ASE2 | <--- | ASE | .965 |
| ASE1 | <--- | ASE | .937 |
| AIL4 | <--- | AIL | .630 |
| AIL3 | <--- | AIL | .759 |
| AIL2 | <--- | AIL | .839 |
| AIL1 | <--- | AIL | .885 |
| SI3 | <--- | SI | .725 |
| SI2 | <--- | SI | .804 |
| SI1 | <--- | SI | .906 |
| TR3 | <--- | TR | .660 |
| TR2 | <--- | TR | .615 |
| TR1 | <--- | TR | .819 |
| AS1 | <--- | ASS | .764 |
| AS2 | <--- | ASS | .713 |
| AS3 | <--- | ASS | .672 |
| AS4 | <--- | ASS | .788 |
| AID1 | <--- | AID | .862 |
| AID2 | <--- | AID | .831 |
| AID3 | <--- | AID | .819 |
| AID4 | <--- | AID | .812 |
| AID5 | <--- | AID | .704 |
| AS5 | <--- | ASS | .613 |
| AID6 | <--- | AID | .499 |
| AID7 | <--- | AID | .513 |
| AIL5 | <--- | AIL | .527 |

## Intercepts: (Group number 1 - Default model)

|  |  |  | Estimate | S.E. | C.R. | P | Label |
| --- | --- | --- | --- | --- | --- | --- | --- |
| ASE5 |  |  | 3.638 | .064 | 57.232 | \*\*\* | par\_42 |
| ASE4 |  |  | 3.725 | .054 | 69.112 | \*\*\* | par\_43 |
| ASE3 |  |  | 3.537 | .058 | 60.761 | \*\*\* | par\_44 |
| ASE2 |  |  | 3.847 | .052 | 74.388 | \*\*\* | par\_45 |
| ASE1 |  |  | 3.790 | .054 | 69.806 | \*\*\* | par\_46 |
| AIL4 |  |  | 3.838 | .056 | 68.541 | \*\*\* | par\_47 |
| AIL3 |  |  | 3.913 | .051 | 76.036 | \*\*\* | par\_48 |
| AIL2 |  |  | 3.834 | .047 | 81.449 | \*\*\* | par\_49 |
| AIL1 |  |  | 3.838 | .050 | 77.216 | \*\*\* | par\_50 |
| SI3 |  |  | 4.048 | .048 | 84.273 | \*\*\* | par\_51 |
| SI2 |  |  | 3.716 | .048 | 77.988 | \*\*\* | par\_52 |
| SI1 |  |  | 3.769 | .046 | 81.102 | \*\*\* | par\_53 |
| TR3 |  |  | 3.428 | .057 | 59.714 | \*\*\* | par\_54 |
| TR2 |  |  | 2.812 | .063 | 44.671 | \*\*\* | par\_55 |
| TR1 |  |  | 3.218 | .057 | 56.223 | \*\*\* | par\_56 |
| AS1 |  |  | 3.039 | .058 | 52.854 | \*\*\* | par\_57 |
| AS2 |  |  | 2.725 | .064 | 42.596 | \*\*\* | par\_58 |
| AS3 |  |  | 2.878 | .062 | 46.430 | \*\*\* | par\_59 |
| AS4 |  |  | 2.795 | .056 | 49.965 | \*\*\* | par\_60 |
| AID1 |  |  | 2.860 | .059 | 48.822 | \*\*\* | par\_61 |
| AID2 |  |  | 3.013 | .062 | 48.380 | \*\*\* | par\_62 |
| AID3 |  |  | 2.930 | .062 | 47.515 | \*\*\* | par\_63 |
| AID4 |  |  | 2.668 | .063 | 42.436 | \*\*\* | par\_64 |
| AID5 |  |  | 2.900 | .060 | 48.700 | \*\*\* | par\_65 |
| AS5 |  |  | 3.301 | .069 | 47.689 | \*\*\* | par\_66 |
| AID6 |  |  | 3.087 | .059 | 52.084 | \*\*\* | par\_67 |
| AID7 |  |  | 3.253 | .059 | 55.333 | \*\*\* | par\_68 |
| AIL5 |  |  | 3.594 | .055 | 65.911 | \*\*\* | par\_69 |

## Covariances: (Group number 1 - Default model)

|  |  |  | Estimate | S.E. | C.R. | P | Label |
| --- | --- | --- | --- | --- | --- | --- | --- |
| ASE | <--> | AIL | .209 | .037 | 5.705 | \*\*\* | par\_26 |
| SI | <--> | ASE | .189 | .033 | 5.743 | \*\*\* | par\_27 |
| SI | <--> | AIL | .177 | .030 | 5.800 | \*\*\* | par\_28 |
| e6 | <--> | e7 | .152 | .027 | 5.548 | \*\*\* | par\_32 |
| e2 | <--> | e3 | .112 | .021 | 5.192 | \*\*\* | par\_33 |
| e24 | <--> | e29 | .173 | .038 | 4.529 | \*\*\* | par\_34 |
| e21 | <--> | e22 | .167 | .037 | 4.503 | \*\*\* | par\_35 |
| e20 | <--> | e23 | .032 | .034 | .941 | .347 | par\_36 |
| e28 | <--> | e29 | .238 | .045 | 5.315 | \*\*\* | par\_37 |
| e6 | <--> | e30 | .100 | .029 | 3.467 | \*\*\* | par\_38 |
| e1 | <--> | e3 | .137 | .030 | 4.526 | \*\*\* | par\_39 |
| e24 | <--> | e28 | .193 | .039 | 4.909 | \*\*\* | par\_40 |
| e1 | <--> | e2 | .096 | .026 | 3.656 | \*\*\* | par\_41 |

## Correlations: (Group number 1 - Default model)

|  |  |  | Estimate |
| --- | --- | --- | --- |
| ASE | <--> | AIL | .627 |
| SI | <--> | ASE | .575 |
| SI | <--> | AIL | .632 |
| e6 | <--> | e7 | .457 |
| e2 | <--> | e3 | .410 |
| e24 | <--> | e29 | .356 |
| e21 | <--> | e22 | .599 |
| e20 | <--> | e23 | .130 |
| e28 | <--> | e29 | .403 |
| e6 | <--> | e30 | .218 |
| e1 | <--> | e3 | .337 |
| e24 | <--> | e28 | .389 |
| e1 | <--> | e2 | .269 |

## Variances: (Group number 1 - Default model)

|  |  |  | Estimate | S.E. | C.R. | P | Label |
| --- | --- | --- | --- | --- | --- | --- | --- |
| ASE |  |  | .391 | .072 | 5.444 | \*\*\* | par\_70 |
| AIL |  |  | .283 | .056 | 5.033 | \*\*\* | par\_71 |
| SI |  |  | .276 | .046 | 6.055 | \*\*\* | par\_72 |
| e26 |  |  | .439 | .069 | 6.325 | \*\*\* | par\_73 |
| e31 |  |  | .274 | .058 | 4.714 | \*\*\* | par\_74 |
| e27 |  |  | .488 | .068 | 7.171 | \*\*\* | par\_75 |
| e1 |  |  | .530 | .051 | 10.312 | \*\*\* | par\_76 |
| e2 |  |  | .238 | .024 | 9.795 | \*\*\* | par\_77 |
| e3 |  |  | .312 | .031 | 9.944 | \*\*\* | par\_78 |
| e4 |  |  | .042 | .011 | 3.750 | \*\*\* | par\_79 |
| e5 |  |  | .082 | .013 | 6.145 | \*\*\* | par\_80 |
| e6 |  |  | .432 | .043 | 9.934 | \*\*\* | par\_81 |
| e7 |  |  | .256 | .029 | 8.919 | \*\*\* | par\_82 |
| e8 |  |  | .149 | .020 | 7.468 | \*\*\* | par\_83 |
| e9 |  |  | .122 | .020 | 5.967 | \*\*\* | par\_84 |
| e10 |  |  | .250 | .027 | 9.124 | \*\*\* | par\_85 |
| e11 |  |  | .183 | .023 | 7.886 | \*\*\* | par\_86 |
| e12 |  |  | .088 | .020 | 4.475 | \*\*\* | par\_87 |
| e13 |  |  | .424 | .054 | 7.897 | \*\*\* | par\_88 |
| e14 |  |  | .561 | .065 | 8.587 | \*\*\* | par\_89 |
| e15 |  |  | .245 | .058 | 4.245 | \*\*\* | par\_90 |
| e16 |  |  | .314 | .040 | 7.894 | \*\*\* | par\_91 |
| e17 |  |  | .459 | .053 | 8.643 | \*\*\* | par\_92 |
| e18 |  |  | .481 | .053 | 9.070 | \*\*\* | par\_93 |
| e19 |  |  | .271 | .036 | 7.435 | \*\*\* | par\_94 |
| e20 |  |  | .201 | .037 | 5.395 | \*\*\* | par\_95 |
| e21 |  |  | .273 | .041 | 6.625 | \*\*\* | par\_96 |
| e22 |  |  | .285 | .041 | 6.904 | \*\*\* | par\_97 |
| e23 |  |  | .307 | .047 | 6.585 | \*\*\* | par\_98 |
| e24 |  |  | .408 | .044 | 9.271 | \*\*\* | par\_99 |
| e25 |  |  | .682 | .072 | 9.506 | \*\*\* | par\_100 |
| e28 |  |  | .601 | .059 | 10.185 | \*\*\* | par\_101 |
| e29 |  |  | .581 | .057 | 10.149 | \*\*\* | par\_102 |
| e30 |  |  | .490 | .048 | 10.198 | \*\*\* | par\_103 |

## Squared Multiple Correlations: (Group number 1 - Default model)

|  |  |  | Estimate |
| --- | --- | --- | --- |
| ASS |  |  | .002 |
| TR |  |  | .163 |
| AID |  |  | .161 |
| AIL5 |  |  | .278 |
| AID7 |  |  | .263 |
| AID6 |  |  | .249 |
| AS5 |  |  | .376 |
| AID5 |  |  | .495 |
| AID4 |  |  | .659 |
| AID3 |  |  | .671 |
| AID2 |  |  | .691 |
| AID1 |  |  | .744 |
| AS4 |  |  | .620 |
| AS3 |  |  | .451 |
| AS2 |  |  | .508 |
| AS1 |  |  | .584 |
| TR1 |  |  | .671 |
| TR2 |  |  | .379 |
| TR3 |  |  | .436 |
| SI1 |  |  | .822 |
| SI2 |  |  | .647 |
| SI3 |  |  | .525 |
| AIL1 |  |  | .783 |
| AIL2 |  |  | .705 |
| AIL3 |  |  | .577 |
| AIL4 |  |  | .396 |
| ASE1 |  |  | .879 |
| ASE2 |  |  | .932 |
| ASE3 |  |  | .597 |
| ASE4 |  |  | .641 |
| ASE5 |  |  | .424 |

## Matrices (Group number 1 - Default model)

## Total Effects (Group number 1 - Default model)

|  | AIL | ASE | SI | ASS | TR | AID |
| --- | --- | --- | --- | --- | --- | --- |
| ASS | .000 | .053 | .000 | .000 | .000 | .000 |
| TR | .000 | .000 | .440 | .000 | .000 | .000 |
| AID | -.281 | .018 | .300 | .351 | .282 | .000 |
| AIL5 | .815 | .000 | .000 | .000 | .000 | .000 |
| AID7 | -.168 | .011 | .179 | .210 | .169 | .597 |
| AID6 | -.165 | .011 | .176 | .206 | .165 | .586 |
| AS5 | .000 | .051 | .000 | .966 | .000 | .000 |
| AID5 | -.233 | .015 | .249 | .292 | .234 | .830 |
| AID4 | -.284 | .019 | .303 | .355 | .285 | 1.011 |
| AID3 | -.281 | .018 | .300 | .351 | .282 | 1.000 |
| AID2 | -.288 | .019 | .308 | .360 | .289 | 1.025 |
| AID1 | -.281 | .018 | .300 | .351 | .282 | 1.000 |
| AS4 | .000 | .053 | .000 | 1.003 | .000 | .000 |
| AS3 | .000 | .050 | .000 | .947 | .000 | .000 |
| AS2 | .000 | .055 | .000 | 1.038 | .000 | .000 |
| AS1 | .000 | .053 | .000 | 1.000 | .000 | .000 |
| TR1 | .000 | .000 | .544 | .000 | 1.238 | .000 |
| TR2 | .000 | .000 | .449 | .000 | 1.022 | .000 |
| TR3 | .000 | .000 | .440 | .000 | 1.000 | .000 |
| SI1 | .000 | .000 | 1.210 | .000 | .000 | .000 |
| SI2 | .000 | .000 | 1.101 | .000 | .000 | .000 |
| SI3 | .000 | .000 | 1.000 | .000 | .000 | .000 |
| AIL1 | 1.248 | .000 | .000 | .000 | .000 | .000 |
| AIL2 | 1.121 | .000 | .000 | .000 | .000 | .000 |
| AIL3 | 1.108 | .000 | .000 | .000 | .000 | .000 |
| AIL4 | 1.000 | .000 | .000 | .000 | .000 | .000 |
| ASE1 | .000 | 1.229 | .000 | .000 | .000 | .000 |
| ASE2 | .000 | 1.206 | .000 | .000 | .000 | .000 |
| ASE3 | .000 | 1.086 | .000 | .000 | .000 | .000 |
| ASE4 | .000 | 1.042 | .000 | .000 | .000 | .000 |
| ASE5 | .000 | 1.000 | .000 | .000 | .000 | .000 |

## Standardized Total Effects (Group number 1 - Default model)

|  | AIL | ASE | SI | ASS | TR | AID |
| --- | --- | --- | --- | --- | --- | --- |
| ASS | .000 | .050 | .000 | .000 | .000 | .000 |
| TR | .000 | .000 | .404 | .000 | .000 | .000 |
| AID | -.196 | .015 | .207 | .306 | .212 | .000 |
| AIL5 | .527 | .000 | .000 | .000 | .000 | .000 |
| AID7 | -.101 | .008 | .106 | .157 | .109 | .513 |
| AID6 | -.098 | .008 | .103 | .153 | .106 | .499 |
| AS5 | .000 | .030 | .000 | .613 | .000 | .000 |
| AID5 | -.138 | .011 | .146 | .215 | .149 | .704 |
| AID4 | -.159 | .012 | .168 | .248 | .172 | .812 |
| AID3 | -.161 | .012 | .170 | .250 | .174 | .819 |
| AID2 | -.163 | .013 | .172 | .254 | .176 | .831 |
| AID1 | -.169 | .013 | .178 | .264 | .183 | .862 |
| AS4 | .000 | .039 | .000 | .788 | .000 | .000 |
| AS3 | .000 | .033 | .000 | .672 | .000 | .000 |
| AS2 | .000 | .035 | .000 | .713 | .000 | .000 |
| AS1 | .000 | .038 | .000 | .764 | .000 | .000 |
| TR1 | .000 | .000 | .331 | .000 | .819 | .000 |
| TR2 | .000 | .000 | .249 | .000 | .615 | .000 |
| TR3 | .000 | .000 | .267 | .000 | .660 | .000 |
| SI1 | .000 | .000 | .906 | .000 | .000 | .000 |
| SI2 | .000 | .000 | .804 | .000 | .000 | .000 |
| SI3 | .000 | .000 | .725 | .000 | .000 | .000 |
| AIL1 | .885 | .000 | .000 | .000 | .000 | .000 |
| AIL2 | .839 | .000 | .000 | .000 | .000 | .000 |
| AIL3 | .759 | .000 | .000 | .000 | .000 | .000 |
| AIL4 | .630 | .000 | .000 | .000 | .000 | .000 |
| ASE1 | .000 | .937 | .000 | .000 | .000 | .000 |
| ASE2 | .000 | .965 | .000 | .000 | .000 | .000 |
| ASE3 | .000 | .772 | .000 | .000 | .000 | .000 |
| ASE4 | .000 | .801 | .000 | .000 | .000 | .000 |
| ASE5 | .000 | .651 | .000 | .000 | .000 | .000 |

## Direct Effects (Group number 1 - Default model)

|  | AIL | ASE | SI | ASS | TR | AID |
| --- | --- | --- | --- | --- | --- | --- |
| ASS | .000 | .053 | .000 | .000 | .000 | .000 |
| TR | .000 | .000 | .440 | .000 | .000 | .000 |
| AID | -.281 | .000 | .176 | .351 | .282 | .000 |
| AIL5 | .815 | .000 | .000 | .000 | .000 | .000 |
| AID7 | .000 | .000 | .000 | .000 | .000 | .597 |
| AID6 | .000 | .000 | .000 | .000 | .000 | .586 |
| AS5 | .000 | .000 | .000 | .966 | .000 | .000 |
| AID5 | .000 | .000 | .000 | .000 | .000 | .830 |
| AID4 | .000 | .000 | .000 | .000 | .000 | 1.011 |
| AID3 | .000 | .000 | .000 | .000 | .000 | 1.000 |
| AID2 | .000 | .000 | .000 | .000 | .000 | 1.025 |
| AID1 | .000 | .000 | .000 | .000 | .000 | 1.000 |
| AS4 | .000 | .000 | .000 | 1.003 | .000 | .000 |
| AS3 | .000 | .000 | .000 | .947 | .000 | .000 |
| AS2 | .000 | .000 | .000 | 1.038 | .000 | .000 |
| AS1 | .000 | .000 | .000 | 1.000 | .000 | .000 |
| TR1 | .000 | .000 | .000 | .000 | 1.238 | .000 |
| TR2 | .000 | .000 | .000 | .000 | 1.022 | .000 |
| TR3 | .000 | .000 | .000 | .000 | 1.000 | .000 |
| SI1 | .000 | .000 | 1.210 | .000 | .000 | .000 |
| SI2 | .000 | .000 | 1.101 | .000 | .000 | .000 |
| SI3 | .000 | .000 | 1.000 | .000 | .000 | .000 |
| AIL1 | 1.248 | .000 | .000 | .000 | .000 | .000 |
| AIL2 | 1.121 | .000 | .000 | .000 | .000 | .000 |
| AIL3 | 1.108 | .000 | .000 | .000 | .000 | .000 |
| AIL4 | 1.000 | .000 | .000 | .000 | .000 | .000 |
| ASE1 | .000 | 1.229 | .000 | .000 | .000 | .000 |
| ASE2 | .000 | 1.206 | .000 | .000 | .000 | .000 |
| ASE3 | .000 | 1.086 | .000 | .000 | .000 | .000 |
| ASE4 | .000 | 1.042 | .000 | .000 | .000 | .000 |
| ASE5 | .000 | 1.000 | .000 | .000 | .000 | .000 |

## Standardized Direct Effects (Group number 1 - Default model)

|  | AIL | ASE | SI | ASS | TR | AID |
| --- | --- | --- | --- | --- | --- | --- |
| ASS | .000 | .050 | .000 | .000 | .000 | .000 |
| TR | .000 | .000 | .404 | .000 | .000 | .000 |
| AID | -.196 | .000 | .121 | .306 | .212 | .000 |
| AIL5 | .527 | .000 | .000 | .000 | .000 | .000 |
| AID7 | .000 | .000 | .000 | .000 | .000 | .513 |
| AID6 | .000 | .000 | .000 | .000 | .000 | .499 |
| AS5 | .000 | .000 | .000 | .613 | .000 | .000 |
| AID5 | .000 | .000 | .000 | .000 | .000 | .704 |
| AID4 | .000 | .000 | .000 | .000 | .000 | .812 |
| AID3 | .000 | .000 | .000 | .000 | .000 | .819 |
| AID2 | .000 | .000 | .000 | .000 | .000 | .831 |
| AID1 | .000 | .000 | .000 | .000 | .000 | .862 |
| AS4 | .000 | .000 | .000 | .788 | .000 | .000 |
| AS3 | .000 | .000 | .000 | .672 | .000 | .000 |
| AS2 | .000 | .000 | .000 | .713 | .000 | .000 |
| AS1 | .000 | .000 | .000 | .764 | .000 | .000 |
| TR1 | .000 | .000 | .000 | .000 | .819 | .000 |
| TR2 | .000 | .000 | .000 | .000 | .615 | .000 |
| TR3 | .000 | .000 | .000 | .000 | .660 | .000 |
| SI1 | .000 | .000 | .906 | .000 | .000 | .000 |
| SI2 | .000 | .000 | .804 | .000 | .000 | .000 |
| SI3 | .000 | .000 | .725 | .000 | .000 | .000 |
| AIL1 | .885 | .000 | .000 | .000 | .000 | .000 |
| AIL2 | .839 | .000 | .000 | .000 | .000 | .000 |
| AIL3 | .759 | .000 | .000 | .000 | .000 | .000 |
| AIL4 | .630 | .000 | .000 | .000 | .000 | .000 |
| ASE1 | .000 | .937 | .000 | .000 | .000 | .000 |
| ASE2 | .000 | .965 | .000 | .000 | .000 | .000 |
| ASE3 | .000 | .772 | .000 | .000 | .000 | .000 |
| ASE4 | .000 | .801 | .000 | .000 | .000 | .000 |
| ASE5 | .000 | .651 | .000 | .000 | .000 | .000 |

## Indirect Effects (Group number 1 - Default model)

|  | AIL | ASE | SI | ASS | TR | AID |
| --- | --- | --- | --- | --- | --- | --- |
| ASS | .000 | .000 | .000 | .000 | .000 | .000 |
| TR | .000 | .000 | .000 | .000 | .000 | .000 |
| AID | .000 | .018 | .124 | .000 | .000 | .000 |
| AIL5 | .000 | .000 | .000 | .000 | .000 | .000 |
| AID7 | -.168 | .011 | .179 | .210 | .169 | .000 |
| AID6 | -.165 | .011 | .176 | .206 | .165 | .000 |
| AS5 | .000 | .051 | .000 | .000 | .000 | .000 |
| AID5 | -.233 | .015 | .249 | .292 | .234 | .000 |
| AID4 | -.284 | .019 | .303 | .355 | .285 | .000 |
| AID3 | -.281 | .018 | .300 | .351 | .282 | .000 |
| AID2 | -.288 | .019 | .308 | .360 | .289 | .000 |
| AID1 | -.281 | .018 | .300 | .351 | .282 | .000 |
| AS4 | .000 | .053 | .000 | .000 | .000 | .000 |
| AS3 | .000 | .050 | .000 | .000 | .000 | .000 |
| AS2 | .000 | .055 | .000 | .000 | .000 | .000 |
| AS1 | .000 | .053 | .000 | .000 | .000 | .000 |
| TR1 | .000 | .000 | .544 | .000 | .000 | .000 |
| TR2 | .000 | .000 | .449 | .000 | .000 | .000 |
| TR3 | .000 | .000 | .440 | .000 | .000 | .000 |
| SI1 | .000 | .000 | .000 | .000 | .000 | .000 |
| SI2 | .000 | .000 | .000 | .000 | .000 | .000 |
| SI3 | .000 | .000 | .000 | .000 | .000 | .000 |
| AIL1 | .000 | .000 | .000 | .000 | .000 | .000 |
| AIL2 | .000 | .000 | .000 | .000 | .000 | .000 |
| AIL3 | .000 | .000 | .000 | .000 | .000 | .000 |
| AIL4 | .000 | .000 | .000 | .000 | .000 | .000 |
| ASE1 | .000 | .000 | .000 | .000 | .000 | .000 |
| ASE2 | .000 | .000 | .000 | .000 | .000 | .000 |
| ASE3 | .000 | .000 | .000 | .000 | .000 | .000 |
| ASE4 | .000 | .000 | .000 | .000 | .000 | .000 |
| ASE5 | .000 | .000 | .000 | .000 | .000 | .000 |

## Standardized Indirect Effects (Group number 1 - Default model)

|  | AIL | ASE | SI | ASS | TR | AID |
| --- | --- | --- | --- | --- | --- | --- |
| ASS | .000 | .000 | .000 | .000 | .000 | .000 |
| TR | .000 | .000 | .000 | .000 | .000 | .000 |
| AID | .000 | .015 | .086 | .000 | .000 | .000 |
| AIL5 | .000 | .000 | .000 | .000 | .000 | .000 |
| AID7 | -.101 | .008 | .106 | .157 | .109 | .000 |
| AID6 | -.098 | .008 | .103 | .153 | .106 | .000 |
| AS5 | .000 | .030 | .000 | .000 | .000 | .000 |
| AID5 | -.138 | .011 | .146 | .215 | .149 | .000 |
| AID4 | -.159 | .012 | .168 | .248 | .172 | .000 |
| AID3 | -.161 | .012 | .170 | .250 | .174 | .000 |
| AID2 | -.163 | .013 | .172 | .254 | .176 | .000 |
| AID1 | -.169 | .013 | .178 | .264 | .183 | .000 |
| AS4 | .000 | .039 | .000 | .000 | .000 | .000 |
| AS3 | .000 | .033 | .000 | .000 | .000 | .000 |
| AS2 | .000 | .035 | .000 | .000 | .000 | .000 |
| AS1 | .000 | .038 | .000 | .000 | .000 | .000 |
| TR1 | .000 | .000 | .331 | .000 | .000 | .000 |
| TR2 | .000 | .000 | .249 | .000 | .000 | .000 |
| TR3 | .000 | .000 | .267 | .000 | .000 | .000 |
| SI1 | .000 | .000 | .000 | .000 | .000 | .000 |
| SI2 | .000 | .000 | .000 | .000 | .000 | .000 |
| SI3 | .000 | .000 | .000 | .000 | .000 | .000 |
| AIL1 | .000 | .000 | .000 | .000 | .000 | .000 |
| AIL2 | .000 | .000 | .000 | .000 | .000 | .000 |
| AIL3 | .000 | .000 | .000 | .000 | .000 | .000 |
| AIL4 | .000 | .000 | .000 | .000 | .000 | .000 |
| ASE1 | .000 | .000 | .000 | .000 | .000 | .000 |
| ASE2 | .000 | .000 | .000 | .000 | .000 | .000 |
| ASE3 | .000 | .000 | .000 | .000 | .000 | .000 |
| ASE4 | .000 | .000 | .000 | .000 | .000 | .000 |
| ASE5 | .000 | .000 | .000 | .000 | .000 | .000 |

## Modification Indices (Group number 1 - Default model)

## Covariances: (Group number 1 - Default model)

|  |  |  | M.I. | Par Change |
| --- | --- | --- | --- | --- |
| e26 | <--> | SI | 5.221 | .048 |
| e31 | <--> | e26 | 9.105 | .088 |
| e30 | <--> | ASE | 5.396 | .053 |
| e30 | <--> | e31 | 4.338 | .058 |
| e29 | <--> | SI | 5.596 | .047 |
| e28 | <--> | e26 | 8.198 | .093 |
| e28 | <--> | e30 | 6.999 | -.082 |
| e25 | <--> | AIL | 5.208 | .056 |
| e25 | <--> | ASE | 4.765 | -.062 |
| e25 | <--> | SI | 6.120 | .063 |
| e25 | <--> | e31 | 6.709 | -.091 |
| e25 | <--> | e27 | 7.454 | .119 |
| e25 | <--> | e30 | 5.232 | -.090 |
| e24 | <--> | ASE | 4.842 | -.043 |
| e24 | <--> | SI | 4.779 | .038 |
| e24 | <--> | e30 | 5.775 | .065 |
| e23 | <--> | e26 | 4.927 | -.063 |
| e22 | <--> | AIL | 6.202 | .031 |
| e22 | <--> | e30 | 4.575 | -.043 |
| e21 | <--> | SI | 6.322 | .032 |
| e19 | <--> | e25 | 6.069 | -.083 |
| e18 | <--> | e29 | 5.329 | -.077 |
| e18 | <--> | e28 | 6.049 | .082 |
| e18 | <--> | e25 | 7.385 | .115 |
| e17 | <--> | e31 | 6.579 | .077 |
| e17 | <--> | e19 | 6.484 | .072 |
| e15 | <--> | ASE | 4.229 | -.045 |
| e15 | <--> | e29 | 12.594 | -.106 |
| e15 | <--> | e24 | 16.591 | .105 |
| e14 | <--> | SI | 9.781 | -.073 |
| e14 | <--> | e27 | 5.807 | .097 |
| e14 | <--> | e25 | 4.074 | -.093 |
| e14 | <--> | e24 | 4.738 | -.068 |
| e14 | <--> | e23 | 4.735 | .069 |
| e14 | <--> | e20 | 4.043 | .055 |
| e13 | <--> | ASE | 8.853 | .070 |
| e13 | <--> | e29 | 10.893 | .106 |
| e12 | <--> | e29 | 6.634 | .048 |
| e12 | <--> | e28 | 7.595 | -.051 |
| e12 | <--> | e20 | 8.514 | -.041 |
| e10 | <--> | e31 | 6.754 | -.056 |
| e10 | <--> | e25 | 16.759 | .125 |
| e10 | <--> | e23 | 4.716 | -.046 |
| e10 | <--> | e21 | 4.846 | .034 |
| e10 | <--> | e14 | 6.987 | -.075 |
| e9 | <--> | e27 | 4.300 | .046 |
| e9 | <--> | e25 | 8.748 | .075 |
| e9 | <--> | e20 | 4.605 | .032 |
| e9 | <--> | e10 | 11.900 | .054 |
| e8 | <--> | AIL | 5.173 | .028 |
| e8 | <--> | ASE | 7.044 | -.039 |
| e7 | <--> | e23 | 15.805 | -.074 |
| e6 | <--> | e25 | 5.059 | -.073 |
| e5 | <--> | e26 | 4.528 | -.034 |
| e5 | <--> | e21 | 4.216 | -.020 |
| e5 | <--> | e16 | 4.460 | .030 |
| e4 | <--> | e18 | 4.252 | -.031 |
| e3 | <--> | e25 | 8.955 | -.085 |
| e3 | <--> | e18 | 7.531 | .067 |
| e3 | <--> | e12 | 12.803 | .049 |
| e3 | <--> | e10 | 9.808 | -.055 |
| e2 | <--> | e12 | 9.151 | -.037 |
| e2 | <--> | e11 | 5.915 | .034 |
| e2 | <--> | e10 | 7.042 | .042 |
| e1 | <--> | e8 | 4.183 | .041 |

## Variances: (Group number 1 - Default model)

|  |  |  | M.I. | Par Change |
| --- | --- | --- | --- | --- |

## Regression Weights: (Group number 1 - Default model)

|  |  |  | M.I. | Par Change |
| --- | --- | --- | --- | --- |
| ASS | <--- | SI | 5.445 | .224 |
| ASS | <--- | TR | 13.487 | .345 |
| TR | <--- | ASS | 9.439 | .204 |
| AIL5 | <--- | TR | 4.492 | .191 |
| AID7 | <--- | SI | 6.831 | .237 |
| AID6 | <--- | ASS | 8.446 | .214 |
| AS5 | <--- | AIL | 9.843 | .357 |
| AS5 | <--- | SI | 11.158 | .386 |
| AS5 | <--- | AID | 4.230 | .164 |
| AID5 | <--- | AIL | 6.117 | -.192 |
| AID5 | <--- | ASE | 7.513 | -.175 |
| AID4 | <--- | ASS | 4.811 | -.142 |
| AS2 | <--- | AIL | 4.519 | -.206 |
| AS2 | <--- | ASE | 5.962 | -.195 |
| AS2 | <--- | SI | 8.423 | -.285 |
| TR2 | <--- | SI | 4.162 | -.219 |
| TR2 | <--- | AID | 4.410 | .155 |
| TR3 | <--- | AIL | 5.247 | .215 |
| TR3 | <--- | ASE | 14.055 | .289 |
| TR3 | <--- | SI | 5.257 | .218 |
| SI3 | <--- | TR | 5.284 | -.160 |
| AIL1 | <--- | AID | 7.680 | .112 |
| AIL2 | <--- | ASE | 5.391 | -.112 |
| ASE1 | <--- | ASS | 4.514 | -.078 |
| ASE1 | <--- | AID | 4.710 | -.068 |

## Means: (Group number 1 - Default model)

|  |  |  | M.I. | Par Change |
| --- | --- | --- | --- | --- |

## Intercepts: (Group number 1 - Default model)

|  |  |  | M.I. | Par Change |
| --- | --- | --- | --- | --- |

## Minimization History (Default model)

| Iteration |  | Negative eigenvalues | Condition # | Smallest eigenvalue | Diameter | F | NTries | Ratio |
| --- | --- | --- | --- | --- | --- | --- | --- | --- |
| 0 | e | 21 |  | -1.278 | 9999.000 | 4374.152 | 0 | 9999.000 |
| 1 | e | 21 |  | -1.521 | 2.512 | 2710.691 | 20 | .531 |
| 2 | e\* | 16 |  | -.322 | .444 | 2339.100 | 6 | .962 |
| 3 | e | 11 |  | -.435 | .613 | 1952.850 | 5 | .883 |
| 4 | e\* | 3 |  | -.129 | .979 | 1311.574 | 5 | .968 |
| 5 | e | 0 | 11234.083 |  | .826 | 1054.412 | 5 | .599 |
| 6 | e | 0 | 4897.138 |  | .723 | 867.640 | 3 | .000 |
| 7 | e | 0 | 2077.410 |  | 1.272 | 709.067 | 1 | 1.021 |
| 8 | e | 0 | 2744.748 |  | .366 | 656.604 | 1 | 1.178 |
| 9 | e | 0 | 2655.767 |  | .193 | 647.942 | 1 | 1.159 |
| 10 | e | 0 | 2638.255 |  | .044 | 647.148 | 1 | 1.080 |
| 11 | e | 0 | 2654.803 |  | .003 | 647.135 | 1 | 1.014 |
| 12 | e | 0 | 2719.473 |  | .000 | 647.135 | 1 | 1.000 |

## Pairwise Parameter Comparisons (Default model)

## Correlations of Estimates (Default model)

|  | c | b | a | par\_4 | par\_5 | par\_6 | par\_7 | par\_8 | par\_9 | par\_10 | par\_11 | par\_12 | par\_13 | par\_14 | par\_15 | par\_16 | par\_17 | par\_18 | par\_19 | par\_20 | par\_21 | par\_22 | par\_23 | par\_24 | par\_25 | par\_26 | par\_27 | par\_28 | par\_29 | par\_30 | par\_31 | par\_32 | par\_33 | par\_34 | par\_35 | par\_36 | par\_37 | par\_38 | par\_39 | par\_40 | par\_41 | par\_42 | par\_43 | par\_44 | par\_45 | par\_46 | par\_47 | par\_48 | par\_49 | par\_50 | par\_51 | par\_52 | par\_53 | par\_54 | par\_55 | par\_56 | par\_57 | par\_58 | par\_59 | par\_60 | par\_61 | par\_62 | par\_63 | par\_64 | par\_65 | par\_66 | par\_67 | par\_68 | par\_69 | par\_70 | par\_71 | par\_72 | par\_73 | par\_74 | par\_75 | par\_76 | par\_77 | par\_78 | par\_79 | par\_80 | par\_81 | par\_82 | par\_83 | par\_84 | par\_85 | par\_86 | par\_87 | par\_88 | par\_89 | par\_90 | par\_91 | par\_92 | par\_93 | par\_94 | par\_95 | par\_96 | par\_97 | par\_98 | par\_99 | par\_100 | par\_101 | par\_102 | par\_103 |
| --- | --- | --- | --- | --- | --- | --- | --- | --- | --- | --- | --- | --- | --- | --- | --- | --- | --- | --- | --- | --- | --- | --- | --- | --- | --- | --- | --- | --- | --- | --- | --- | --- | --- | --- | --- | --- | --- | --- | --- | --- | --- | --- | --- | --- | --- | --- | --- | --- | --- | --- | --- | --- | --- | --- | --- | --- | --- | --- | --- | --- | --- | --- | --- | --- | --- | --- | --- | --- | --- | --- | --- | --- | --- | --- | --- | --- | --- | --- | --- | --- | --- | --- | --- | --- | --- | --- | --- | --- | --- | --- | --- | --- | --- | --- | --- | --- | --- | --- | --- | --- | --- | --- | --- |
| c | 1.000 |  |  |  |  |  |  |  |  |  |  |  |  |  |  |  |  |  |  |  |  |  |  |  |  |  |  |  |  |  |  |  |  |  |  |  |  |  |  |  |  |  |  |  |  |  |  |  |  |  |  |  |  |  |  |  |  |  |  |  |  |  |  |  |  |  |  |  |  |  |  |  |  |  |  |  |  |  |  |  |  |  |  |  |  |  |  |  |  |  |  |  |  |  |  |  |  |  |  |  |  |  |  |
| b | -.363 | 1.000 |  |  |  |  |  |  |  |  |  |  |  |  |  |  |  |  |  |  |  |  |  |  |  |  |  |  |  |  |  |  |  |  |  |  |  |  |  |  |  |  |  |  |  |  |  |  |  |  |  |  |  |  |  |  |  |  |  |  |  |  |  |  |  |  |  |  |  |  |  |  |  |  |  |  |  |  |  |  |  |  |  |  |  |  |  |  |  |  |  |  |  |  |  |  |  |  |  |  |  |  |  |
| a | -.012 | -.082 | 1.000 |  |  |  |  |  |  |  |  |  |  |  |  |  |  |  |  |  |  |  |  |  |  |  |  |  |  |  |  |  |  |  |  |  |  |  |  |  |  |  |  |  |  |  |  |  |  |  |  |  |  |  |  |  |  |  |  |  |  |  |  |  |  |  |  |  |  |  |  |  |  |  |  |  |  |  |  |  |  |  |  |  |  |  |  |  |  |  |  |  |  |  |  |  |  |  |  |  |  |  |  |
| par\_4 | .000 | .000 | .000 | 1.000 |  |  |  |  |  |  |  |  |  |  |  |  |  |  |  |  |  |  |  |  |  |  |  |  |  |  |  |  |  |  |  |  |  |  |  |  |  |  |  |  |  |  |  |  |  |  |  |  |  |  |  |  |  |  |  |  |  |  |  |  |  |  |  |  |  |  |  |  |  |  |  |  |  |  |  |  |  |  |  |  |  |  |  |  |  |  |  |  |  |  |  |  |  |  |  |  |  |  |  |
| par\_5 | .000 | .000 | .000 | .742 | 1.000 |  |  |  |  |  |  |  |  |  |  |  |  |  |  |  |  |  |  |  |  |  |  |  |  |  |  |  |  |  |  |  |  |  |  |  |  |  |  |  |  |  |  |  |  |  |  |  |  |  |  |  |  |  |  |  |  |  |  |  |  |  |  |  |  |  |  |  |  |  |  |  |  |  |  |  |  |  |  |  |  |  |  |  |  |  |  |  |  |  |  |  |  |  |  |  |  |  |  |
| par\_6 | .000 | .000 | .000 | .771 | .726 | 1.000 |  |  |  |  |  |  |  |  |  |  |  |  |  |  |  |  |  |  |  |  |  |  |  |  |  |  |  |  |  |  |  |  |  |  |  |  |  |  |  |  |  |  |  |  |  |  |  |  |  |  |  |  |  |  |  |  |  |  |  |  |  |  |  |  |  |  |  |  |  |  |  |  |  |  |  |  |  |  |  |  |  |  |  |  |  |  |  |  |  |  |  |  |  |  |  |  |  |
| par\_7 | .000 | .000 | .000 | .760 | .715 | .911 | 1.000 |  |  |  |  |  |  |  |  |  |  |  |  |  |  |  |  |  |  |  |  |  |  |  |  |  |  |  |  |  |  |  |  |  |  |  |  |  |  |  |  |  |  |  |  |  |  |  |  |  |  |  |  |  |  |  |  |  |  |  |  |  |  |  |  |  |  |  |  |  |  |  |  |  |  |  |  |  |  |  |  |  |  |  |  |  |  |  |  |  |  |  |  |  |  |  |  |
| par\_8 | .000 | .000 | .000 | .000 | .000 | .000 | .000 | 1.000 |  |  |  |  |  |  |  |  |  |  |  |  |  |  |  |  |  |  |  |  |  |  |  |  |  |  |  |  |  |  |  |  |  |  |  |  |  |  |  |  |  |  |  |  |  |  |  |  |  |  |  |  |  |  |  |  |  |  |  |  |  |  |  |  |  |  |  |  |  |  |  |  |  |  |  |  |  |  |  |  |  |  |  |  |  |  |  |  |  |  |  |  |  |  |  |
| par\_9 | -.001 | .000 | .000 | .000 | .000 | .000 | .000 | .647 | 1.000 |  |  |  |  |  |  |  |  |  |  |  |  |  |  |  |  |  |  |  |  |  |  |  |  |  |  |  |  |  |  |  |  |  |  |  |  |  |  |  |  |  |  |  |  |  |  |  |  |  |  |  |  |  |  |  |  |  |  |  |  |  |  |  |  |  |  |  |  |  |  |  |  |  |  |  |  |  |  |  |  |  |  |  |  |  |  |  |  |  |  |  |  |  |  |
| par\_10 | -.003 | .000 | .000 | .000 | .000 | .000 | .000 | .665 | .787 | 1.000 |  |  |  |  |  |  |  |  |  |  |  |  |  |  |  |  |  |  |  |  |  |  |  |  |  |  |  |  |  |  |  |  |  |  |  |  |  |  |  |  |  |  |  |  |  |  |  |  |  |  |  |  |  |  |  |  |  |  |  |  |  |  |  |  |  |  |  |  |  |  |  |  |  |  |  |  |  |  |  |  |  |  |  |  |  |  |  |  |  |  |  |  |  |
| par\_11 | .061 | .000 | .245 | .000 | .000 | .000 | .000 | .000 | .000 | .000 | 1.000 |  |  |  |  |  |  |  |  |  |  |  |  |  |  |  |  |  |  |  |  |  |  |  |  |  |  |  |  |  |  |  |  |  |  |  |  |  |  |  |  |  |  |  |  |  |  |  |  |  |  |  |  |  |  |  |  |  |  |  |  |  |  |  |  |  |  |  |  |  |  |  |  |  |  |  |  |  |  |  |  |  |  |  |  |  |  |  |  |  |  |  |  |
| par\_12 | .060 | .003 | .266 | .000 | .000 | .000 | .000 | .000 | .000 | .000 | .649 | 1.000 |  |  |  |  |  |  |  |  |  |  |  |  |  |  |  |  |  |  |  |  |  |  |  |  |  |  |  |  |  |  |  |  |  |  |  |  |  |  |  |  |  |  |  |  |  |  |  |  |  |  |  |  |  |  |  |  |  |  |  |  |  |  |  |  |  |  |  |  |  |  |  |  |  |  |  |  |  |  |  |  |  |  |  |  |  |  |  |  |  |  |  |
| par\_13 | .000 | .150 | -.286 | .000 | .000 | .000 | .000 | .000 | .000 | .000 | .000 | .000 | 1.000 |  |  |  |  |  |  |  |  |  |  |  |  |  |  |  |  |  |  |  |  |  |  |  |  |  |  |  |  |  |  |  |  |  |  |  |  |  |  |  |  |  |  |  |  |  |  |  |  |  |  |  |  |  |  |  |  |  |  |  |  |  |  |  |  |  |  |  |  |  |  |  |  |  |  |  |  |  |  |  |  |  |  |  |  |  |  |  |  |  |  |
| par\_14 | .019 | .160 | -.412 | .000 | .000 | .000 | .000 | .000 | .000 | .000 | .000 | .000 | .488 | 1.000 |  |  |  |  |  |  |  |  |  |  |  |  |  |  |  |  |  |  |  |  |  |  |  |  |  |  |  |  |  |  |  |  |  |  |  |  |  |  |  |  |  |  |  |  |  |  |  |  |  |  |  |  |  |  |  |  |  |  |  |  |  |  |  |  |  |  |  |  |  |  |  |  |  |  |  |  |  |  |  |  |  |  |  |  |  |  |  |  |  |
| par\_15 | .000 | .000 | .000 | .000 | .000 | .000 | .000 | .000 | .000 | .000 | .000 | .000 | .000 | .000 | 1.000 |  |  |  |  |  |  |  |  |  |  |  |  |  |  |  |  |  |  |  |  |  |  |  |  |  |  |  |  |  |  |  |  |  |  |  |  |  |  |  |  |  |  |  |  |  |  |  |  |  |  |  |  |  |  |  |  |  |  |  |  |  |  |  |  |  |  |  |  |  |  |  |  |  |  |  |  |  |  |  |  |  |  |  |  |  |  |  |  |
| par\_16 | .000 | .000 | .000 | .000 | .000 | .000 | .000 | .000 | .000 | .000 | .000 | .000 | .000 | .000 | .412 | 1.000 |  |  |  |  |  |  |  |  |  |  |  |  |  |  |  |  |  |  |  |  |  |  |  |  |  |  |  |  |  |  |  |  |  |  |  |  |  |  |  |  |  |  |  |  |  |  |  |  |  |  |  |  |  |  |  |  |  |  |  |  |  |  |  |  |  |  |  |  |  |  |  |  |  |  |  |  |  |  |  |  |  |  |  |  |  |  |  |
| par\_17 | .000 | .000 | .000 | .000 | .000 | .000 | .000 | .000 | .000 | .000 | .000 | .000 | .000 | .000 | .481 | .454 | 1.000 |  |  |  |  |  |  |  |  |  |  |  |  |  |  |  |  |  |  |  |  |  |  |  |  |  |  |  |  |  |  |  |  |  |  |  |  |  |  |  |  |  |  |  |  |  |  |  |  |  |  |  |  |  |  |  |  |  |  |  |  |  |  |  |  |  |  |  |  |  |  |  |  |  |  |  |  |  |  |  |  |  |  |  |  |  |  |
| par\_18 | -.038 | -.081 | .000 | .000 | .000 | .000 | .000 | .000 | .000 | .000 | .000 | .000 | .000 | .000 | .000 | .000 | .000 | 1.000 |  |  |  |  |  |  |  |  |  |  |  |  |  |  |  |  |  |  |  |  |  |  |  |  |  |  |  |  |  |  |  |  |  |  |  |  |  |  |  |  |  |  |  |  |  |  |  |  |  |  |  |  |  |  |  |  |  |  |  |  |  |  |  |  |  |  |  |  |  |  |  |  |  |  |  |  |  |  |  |  |  |  |  |  |  |
| par\_19 | -.037 | -.079 | .000 | .000 | .000 | .000 | .000 | .000 | .000 | .000 | .000 | .000 | .000 | .000 | .000 | .000 | .000 | .836 | 1.000 |  |  |  |  |  |  |  |  |  |  |  |  |  |  |  |  |  |  |  |  |  |  |  |  |  |  |  |  |  |  |  |  |  |  |  |  |  |  |  |  |  |  |  |  |  |  |  |  |  |  |  |  |  |  |  |  |  |  |  |  |  |  |  |  |  |  |  |  |  |  |  |  |  |  |  |  |  |  |  |  |  |  |  |  |
| par\_20 | -.029 | -.060 | .000 | .000 | .000 | .000 | .000 | .000 | .000 | .000 | .000 | .000 | .000 | .000 | .000 | .000 | .000 | .325 | .319 | 1.000 |  |  |  |  |  |  |  |  |  |  |  |  |  |  |  |  |  |  |  |  |  |  |  |  |  |  |  |  |  |  |  |  |  |  |  |  |  |  |  |  |  |  |  |  |  |  |  |  |  |  |  |  |  |  |  |  |  |  |  |  |  |  |  |  |  |  |  |  |  |  |  |  |  |  |  |  |  |  |  |  |  |  |  |
| par\_21 | -.030 | -.063 | .000 | .000 | .000 | .000 | .000 | .000 | .000 | .000 | .000 | .000 | .000 | .000 | .000 | .000 | .000 | .436 | .428 | .279 | 1.000 |  |  |  |  |  |  |  |  |  |  |  |  |  |  |  |  |  |  |  |  |  |  |  |  |  |  |  |  |  |  |  |  |  |  |  |  |  |  |  |  |  |  |  |  |  |  |  |  |  |  |  |  |  |  |  |  |  |  |  |  |  |  |  |  |  |  |  |  |  |  |  |  |  |  |  |  |  |  |  |  |  |  |
| par\_22 | -.010 | .001 | .000 | .043 | .040 | .051 | .051 | .000 | .000 | .000 | .000 | .000 | .000 | .000 | -.029 | -.027 | -.032 | .000 | .000 | .000 | .000 | 1.000 |  |  |  |  |  |  |  |  |  |  |  |  |  |  |  |  |  |  |  |  |  |  |  |  |  |  |  |  |  |  |  |  |  |  |  |  |  |  |  |  |  |  |  |  |  |  |  |  |  |  |  |  |  |  |  |  |  |  |  |  |  |  |  |  |  |  |  |  |  |  |  |  |  |  |  |  |  |  |  |  |  |
| par\_23 | -.003 | .015 | .000 | .000 | .000 | .000 | .000 | .000 | .000 | .000 | .000 | .000 | .000 | .000 | .175 | .165 | .194 | -.135 | -.133 | -.101 | -.106 | -.010 | 1.000 |  |  |  |  |  |  |  |  |  |  |  |  |  |  |  |  |  |  |  |  |  |  |  |  |  |  |  |  |  |  |  |  |  |  |  |  |  |  |  |  |  |  |  |  |  |  |  |  |  |  |  |  |  |  |  |  |  |  |  |  |  |  |  |  |  |  |  |  |  |  |  |  |  |  |  |  |  |  |  |  |
| par\_24 | -.647 | .028 | .000 | .000 | .000 | .000 | .000 | -.128 | -.152 | -.155 | .000 | .008 | .000 | .000 | .000 | .000 | .000 | .066 | .065 | .049 | .052 | -.017 | -.029 | 1.000 |  |  |  |  |  |  |  |  |  |  |  |  |  |  |  |  |  |  |  |  |  |  |  |  |  |  |  |  |  |  |  |  |  |  |  |  |  |  |  |  |  |  |  |  |  |  |  |  |  |  |  |  |  |  |  |  |  |  |  |  |  |  |  |  |  |  |  |  |  |  |  |  |  |  |  |  |  |  |  |
| par\_25 | .000 | .000 | .000 | .000 | .000 | .000 | .000 | .000 | .000 | .000 | .000 | .000 | .000 | .000 | .375 | .353 | .414 | .000 | .000 | .000 | .000 | -.024 | .150 | .000 | 1.000 |  |  |  |  |  |  |  |  |  |  |  |  |  |  |  |  |  |  |  |  |  |  |  |  |  |  |  |  |  |  |  |  |  |  |  |  |  |  |  |  |  |  |  |  |  |  |  |  |  |  |  |  |  |  |  |  |  |  |  |  |  |  |  |  |  |  |  |  |  |  |  |  |  |  |  |  |  |  |
| par\_26 | .004 | .000 | .000 | -.360 | -.339 | -.439 | -.427 | -.369 | -.441 | -.460 | .000 | .000 | .000 | .000 | .000 | .000 | .000 | .000 | .000 | .000 | .000 | -.024 | .000 | .084 | .000 | 1.000 |  |  |  |  |  |  |  |  |  |  |  |  |  |  |  |  |  |  |  |  |  |  |  |  |  |  |  |  |  |  |  |  |  |  |  |  |  |  |  |  |  |  |  |  |  |  |  |  |  |  |  |  |  |  |  |  |  |  |  |  |  |  |  |  |  |  |  |  |  |  |  |  |  |  |  |  |  |
| par\_27 | -.026 | .000 | -.122 | -.363 | -.342 | -.442 | -.430 | .000 | .000 | .000 | -.304 | -.360 | .000 | .000 | .000 | .000 | .000 | .000 | .000 | .000 | .000 | -.025 | .000 | -.006 | .000 | .606 | 1.000 |  |  |  |  |  |  |  |  |  |  |  |  |  |  |  |  |  |  |  |  |  |  |  |  |  |  |  |  |  |  |  |  |  |  |  |  |  |  |  |  |  |  |  |  |  |  |  |  |  |  |  |  |  |  |  |  |  |  |  |  |  |  |  |  |  |  |  |  |  |  |  |  |  |  |  |  |
| par\_28 | -.010 | -.001 | -.123 | .000 | .000 | .000 | .000 | -.376 | -.448 | -.468 | -.307 | -.364 | .000 | .000 | .000 | .000 | .000 | .000 | .000 | .000 | .000 | .000 | .000 | .068 | .000 | .619 | .571 | 1.000 |  |  |  |  |  |  |  |  |  |  |  |  |  |  |  |  |  |  |  |  |  |  |  |  |  |  |  |  |  |  |  |  |  |  |  |  |  |  |  |  |  |  |  |  |  |  |  |  |  |  |  |  |  |  |  |  |  |  |  |  |  |  |  |  |  |  |  |  |  |  |  |  |  |  |  |
| par\_29 | -.020 | -.042 | .000 | .000 | .000 | .000 | .000 | .000 | .000 | .000 | .000 | .000 | .000 | .000 | .000 | .000 | .000 | .290 | .285 | .186 | .523 | .000 | -.070 | .034 | .000 | .000 | .000 | .000 | 1.000 |  |  |  |  |  |  |  |  |  |  |  |  |  |  |  |  |  |  |  |  |  |  |  |  |  |  |  |  |  |  |  |  |  |  |  |  |  |  |  |  |  |  |  |  |  |  |  |  |  |  |  |  |  |  |  |  |  |  |  |  |  |  |  |  |  |  |  |  |  |  |  |  |  |  |
| par\_30 | -.020 | -.043 | .000 | .000 | .000 | .000 | .000 | .000 | .000 | .000 | .000 | .000 | .000 | .000 | .000 | .000 | .000 | .299 | .293 | .191 | .505 | .000 | -.073 | .035 | .000 | .000 | .000 | .000 | .500 | 1.000 |  |  |  |  |  |  |  |  |  |  |  |  |  |  |  |  |  |  |  |  |  |  |  |  |  |  |  |  |  |  |  |  |  |  |  |  |  |  |  |  |  |  |  |  |  |  |  |  |  |  |  |  |  |  |  |  |  |  |  |  |  |  |  |  |  |  |  |  |  |  |  |  |  |
| par\_31 | .000 | .000 | .000 | .000 | .000 | .000 | .000 | .298 | .436 | .449 | .000 | .000 | .000 | .000 | .000 | .000 | .000 | .000 | .000 | .000 | .000 | .000 | .000 | -.086 | .000 | -.248 | .000 | -.252 | .000 | .000 | 1.000 |  |  |  |  |  |  |  |  |  |  |  |  |  |  |  |  |  |  |  |  |  |  |  |  |  |  |  |  |  |  |  |  |  |  |  |  |  |  |  |  |  |  |  |  |  |  |  |  |  |  |  |  |  |  |  |  |  |  |  |  |  |  |  |  |  |  |  |  |  |  |  |  |
| par\_32 | .002 | .000 | .000 | .000 | .000 | .000 | .000 | .067 | .151 | .169 | .000 | .000 | .000 | .000 | .000 | .000 | .000 | .000 | .000 | .000 | .000 | .000 | .000 | -.030 | .000 | -.079 | .000 | -.080 | .000 | .000 | .114 | 1.000 |  |  |  |  |  |  |  |  |  |  |  |  |  |  |  |  |  |  |  |  |  |  |  |  |  |  |  |  |  |  |  |  |  |  |  |  |  |  |  |  |  |  |  |  |  |  |  |  |  |  |  |  |  |  |  |  |  |  |  |  |  |  |  |  |  |  |  |  |  |  |  |
| par\_33 | .000 | .000 | .000 | -.013 | -.019 | .052 | .031 | .000 | .000 | .000 | .000 | .000 | .000 | .000 | .000 | .000 | .000 | .000 | .000 | .000 | .000 | .002 | .000 | .000 | .000 | -.018 | -.018 | .000 | .000 | .000 | .000 | .000 | 1.000 |  |  |  |  |  |  |  |  |  |  |  |  |  |  |  |  |  |  |  |  |  |  |  |  |  |  |  |  |  |  |  |  |  |  |  |  |  |  |  |  |  |  |  |  |  |  |  |  |  |  |  |  |  |  |  |  |  |  |  |  |  |  |  |  |  |  |  |  |  |  |
| par\_34 | .003 | .007 | .000 | .000 | .000 | .000 | .000 | .000 | .000 | .000 | .000 | .000 | .000 | .000 | .000 | .000 | .000 | -.047 | -.046 | .000 | -.139 | .000 | .012 | -.006 | .000 | .000 | .000 | .000 | -.101 | -.159 | .000 | .000 | .000 | 1.000 |  |  |  |  |  |  |  |  |  |  |  |  |  |  |  |  |  |  |  |  |  |  |  |  |  |  |  |  |  |  |  |  |  |  |  |  |  |  |  |  |  |  |  |  |  |  |  |  |  |  |  |  |  |  |  |  |  |  |  |  |  |  |  |  |  |  |  |  |  |
| par\_35 | .016 | .033 | .000 | .000 | .000 | .000 | .000 | .000 | .000 | .000 | .000 | .000 | .000 | .000 | .000 | .000 | .000 | -.456 | -.454 | .000 | -.173 | .000 | .056 | -.027 | .000 | .000 | .000 | .000 | -.115 | -.119 | .000 | .000 | .000 | .014 | 1.000 |  |  |  |  |  |  |  |  |  |  |  |  |  |  |  |  |  |  |  |  |  |  |  |  |  |  |  |  |  |  |  |  |  |  |  |  |  |  |  |  |  |  |  |  |  |  |  |  |  |  |  |  |  |  |  |  |  |  |  |  |  |  |  |  |  |  |  |  |
| par\_36 | -.017 | -.037 | .000 | .000 | .000 | .000 | .000 | .000 | .000 | .000 | .000 | .000 | .000 | .000 | .000 | .000 | .000 | .472 | .463 | -.052 | .312 | .000 | -.061 | .030 | .000 | .000 | .000 | .000 | .208 | .214 | .000 | .000 | .000 | -.109 | -.497 | 1.000 |  |  |  |  |  |  |  |  |  |  |  |  |  |  |  |  |  |  |  |  |  |  |  |  |  |  |  |  |  |  |  |  |  |  |  |  |  |  |  |  |  |  |  |  |  |  |  |  |  |  |  |  |  |  |  |  |  |  |  |  |  |  |  |  |  |  |  |
| par\_37 | .002 | .004 | .000 | .000 | .000 | .000 | .000 | .000 | .000 | .000 | .000 | .000 | .000 | .000 | .000 | .000 | .000 | -.028 | -.028 | .000 | -.074 | .000 | .007 | -.004 | .000 | .000 | .000 | .000 | -.109 | -.108 | .000 | .000 | .000 | .518 | .009 | -.066 | 1.000 |  |  |  |  |  |  |  |  |  |  |  |  |  |  |  |  |  |  |  |  |  |  |  |  |  |  |  |  |  |  |  |  |  |  |  |  |  |  |  |  |  |  |  |  |  |  |  |  |  |  |  |  |  |  |  |  |  |  |  |  |  |  |  |  |  |  |
| par\_38 | .000 | .000 | .000 | .000 | .000 | .000 | .000 | .084 | .070 | .075 | .000 | .000 | .000 | .000 | .000 | .000 | .000 | .000 | .000 | .000 | .000 | .000 | .000 | -.014 | .000 | -.038 | .000 | -.039 | .000 | .000 | .007 | .067 | .000 | .000 | .000 | .000 | .000 | 1.000 |  |  |  |  |  |  |  |  |  |  |  |  |  |  |  |  |  |  |  |  |  |  |  |  |  |  |  |  |  |  |  |  |  |  |  |  |  |  |  |  |  |  |  |  |  |  |  |  |  |  |  |  |  |  |  |  |  |  |  |  |  |  |  |  |  |
| par\_39 | .000 | .000 | .000 | .042 | .024 | .074 | .059 | .000 | .000 | .000 | .000 | .000 | .000 | .000 | .000 | .000 | .000 | .000 | .000 | .000 | .000 | .003 | .000 | .000 | .000 | -.031 | -.031 | .000 | .000 | .000 | .000 | .000 | .410 | .000 | .000 | .000 | .000 | .000 | 1.000 |  |  |  |  |  |  |  |  |  |  |  |  |  |  |  |  |  |  |  |  |  |  |  |  |  |  |  |  |  |  |  |  |  |  |  |  |  |  |  |  |  |  |  |  |  |  |  |  |  |  |  |  |  |  |  |  |  |  |  |  |  |  |  |  |
| par\_40 | .003 | .007 | .000 | .000 | .000 | .000 | .000 | .000 | .000 | .000 | .000 | .000 | .000 | .000 | .000 | .000 | .000 | -.045 | -.044 | .000 | -.137 | .000 | .012 | -.006 | .000 | .000 | .000 | .000 | -.158 | -.097 | .000 | .000 | .000 | .522 | .014 | -.104 | .500 | .000 | .000 | 1.000 |  |  |  |  |  |  |  |  |  |  |  |  |  |  |  |  |  |  |  |  |  |  |  |  |  |  |  |  |  |  |  |  |  |  |  |  |  |  |  |  |  |  |  |  |  |  |  |  |  |  |  |  |  |  |  |  |  |  |  |  |  |  |  |
| par\_41 | .000 | .000 | .000 | .031 | .040 | .079 | .062 | .000 | .000 | .000 | .000 | .000 | .000 | .000 | .000 | .000 | .000 | .000 | .000 | .000 | .000 | .004 | .000 | .000 | .000 | -.032 | -.033 | .000 | .000 | .000 | .000 | .000 | .447 | .000 | .000 | .000 | .000 | .000 | .510 | .000 | 1.000 |  |  |  |  |  |  |  |  |  |  |  |  |  |  |  |  |  |  |  |  |  |  |  |  |  |  |  |  |  |  |  |  |  |  |  |  |  |  |  |  |  |  |  |  |  |  |  |  |  |  |  |  |  |  |  |  |  |  |  |  |  |  |
| par\_42 | .000 | .000 | .000 | .000 | .000 | .000 | .000 | .000 | .000 | .000 | .000 | .000 | .000 | .000 | .000 | .000 | .000 | .000 | .000 | .000 | .000 | .000 | .000 | .000 | .000 | .000 | .000 | .000 | .000 | .000 | .000 | .000 | .000 | .000 | .000 | .000 | .000 | .000 | .000 | .000 | .000 | 1.000 |  |  |  |  |  |  |  |  |  |  |  |  |  |  |  |  |  |  |  |  |  |  |  |  |  |  |  |  |  |  |  |  |  |  |  |  |  |  |  |  |  |  |  |  |  |  |  |  |  |  |  |  |  |  |  |  |  |  |  |  |  |
| par\_43 | .000 | .000 | .000 | .000 | .000 | .000 | .000 | .000 | .000 | .000 | .000 | .000 | .000 | .000 | .000 | .000 | .000 | .000 | .000 | .000 | .000 | .000 | .000 | .000 | .000 | .000 | .000 | .000 | .000 | .000 | .000 | .000 | .000 | .000 | .000 | .000 | .000 | .000 | .000 | .000 | .000 | .644 | 1.000 |  |  |  |  |  |  |  |  |  |  |  |  |  |  |  |  |  |  |  |  |  |  |  |  |  |  |  |  |  |  |  |  |  |  |  |  |  |  |  |  |  |  |  |  |  |  |  |  |  |  |  |  |  |  |  |  |  |  |  |  |
| par\_44 | .000 | .000 | .000 | .000 | .000 | .000 | .000 | .000 | .000 | .000 | .000 | .000 | .000 | .000 | .000 | .000 | .000 | .000 | .000 | .000 | .000 | .000 | .000 | .000 | .000 | .000 | .000 | .000 | .000 | .000 | .000 | .000 | .000 | .000 | .000 | .000 | .000 | .000 | .000 | .000 | .000 | .666 | .774 | 1.000 |  |  |  |  |  |  |  |  |  |  |  |  |  |  |  |  |  |  |  |  |  |  |  |  |  |  |  |  |  |  |  |  |  |  |  |  |  |  |  |  |  |  |  |  |  |  |  |  |  |  |  |  |  |  |  |  |  |  |  |
| par\_45 | .000 | .000 | .000 | .000 | .000 | .000 | .000 | .000 | .000 | .000 | .000 | .000 | .000 | .000 | .000 | .000 | .000 | .000 | .000 | .000 | .000 | .000 | .000 | .000 | .000 | .000 | .000 | .000 | .000 | .000 | .000 | .000 | .000 | .000 | .000 | .000 | .000 | .000 | .000 | .000 | .000 | .629 | .773 | .746 | 1.000 |  |  |  |  |  |  |  |  |  |  |  |  |  |  |  |  |  |  |  |  |  |  |  |  |  |  |  |  |  |  |  |  |  |  |  |  |  |  |  |  |  |  |  |  |  |  |  |  |  |  |  |  |  |  |  |  |  |  |
| par\_46 | .000 | .000 | .000 | .000 | .000 | .000 | .000 | .000 | .000 | .000 | .000 | .000 | .000 | .000 | .000 | .000 | .000 | .000 | .000 | .000 | .000 | .000 | .000 | .000 | .000 | .000 | .000 | .000 | .000 | .000 | .000 | .000 | .000 | .000 | .000 | .000 | .000 | .000 | .000 | .000 | .000 | .611 | .750 | .724 | .905 | 1.000 |  |  |  |  |  |  |  |  |  |  |  |  |  |  |  |  |  |  |  |  |  |  |  |  |  |  |  |  |  |  |  |  |  |  |  |  |  |  |  |  |  |  |  |  |  |  |  |  |  |  |  |  |  |  |  |  |  |
| par\_47 | .000 | .000 | .000 | .000 | .000 | .000 | .000 | .000 | .000 | .000 | .000 | .000 | .000 | .000 | .000 | .000 | .000 | .000 | .000 | .000 | .000 | .000 | .000 | .000 | .000 | .000 | .000 | .000 | .000 | .000 | .000 | .000 | .000 | .000 | .000 | .000 | .000 | .000 | .000 | .000 | .000 | .257 | .316 | .305 | .381 | .370 | 1.000 |  |  |  |  |  |  |  |  |  |  |  |  |  |  |  |  |  |  |  |  |  |  |  |  |  |  |  |  |  |  |  |  |  |  |  |  |  |  |  |  |  |  |  |  |  |  |  |  |  |  |  |  |  |  |  |  |
| par\_48 | .000 | .000 | .000 | .000 | .000 | .000 | .000 | .000 | .000 | .000 | .000 | .000 | .000 | .000 | .000 | .000 | .000 | .000 | .000 | .000 | .000 | .000 | .000 | .000 | .000 | .000 | .000 | .000 | .000 | .000 | .000 | .000 | .000 | .000 | .000 | .000 | .000 | .000 | .000 | .000 | .000 | .310 | .381 | .368 | .459 | .446 | .709 | 1.000 |  |  |  |  |  |  |  |  |  |  |  |  |  |  |  |  |  |  |  |  |  |  |  |  |  |  |  |  |  |  |  |  |  |  |  |  |  |  |  |  |  |  |  |  |  |  |  |  |  |  |  |  |  |  |  |
| par\_49 | .000 | .000 | .000 | .000 | .000 | .000 | .000 | .000 | .000 | .000 | .000 | .000 | .000 | .000 | .000 | .000 | .000 | .000 | .000 | .000 | .000 | .000 | .000 | .000 | .000 | .000 | .000 | .000 | .000 | .000 | .000 | .000 | .000 | .000 | .000 | .000 | .000 | .000 | .000 | .000 | .000 | .343 | .421 | .406 | .508 | .493 | .528 | .637 | 1.000 |  |  |  |  |  |  |  |  |  |  |  |  |  |  |  |  |  |  |  |  |  |  |  |  |  |  |  |  |  |  |  |  |  |  |  |  |  |  |  |  |  |  |  |  |  |  |  |  |  |  |  |  |  |  |
| par\_50 | .000 | .000 | .000 | .000 | .000 | .000 | .000 | .000 | .000 | .000 | .000 | .000 | .000 | .000 | .000 | .000 | .000 | .000 | .000 | .000 | .000 | .000 | .000 | .000 | .000 | .000 | .000 | .000 | .000 | .000 | .000 | .000 | .000 | .000 | .000 | .000 | .000 | .000 | .000 | .000 | .000 | .361 | .444 | .428 | .535 | .520 | .557 | .672 | .743 | 1.000 |  |  |  |  |  |  |  |  |  |  |  |  |  |  |  |  |  |  |  |  |  |  |  |  |  |  |  |  |  |  |  |  |  |  |  |  |  |  |  |  |  |  |  |  |  |  |  |  |  |  |  |  |  |
| par\_51 | .000 | .000 | .000 | .000 | .000 | .000 | .000 | .000 | .000 | .000 | .000 | .000 | .000 | .000 | .000 | .000 | .000 | .000 | .000 | .000 | .000 | .000 | .000 | .000 | .000 | .000 | .000 | .000 | .000 | .000 | .000 | .000 | .000 | .000 | .000 | .000 | .000 | .000 | .000 | .000 | .000 | .271 | .333 | .322 | .402 | .390 | .288 | .348 | .384 | .405 | 1.000 |  |  |  |  |  |  |  |  |  |  |  |  |  |  |  |  |  |  |  |  |  |  |  |  |  |  |  |  |  |  |  |  |  |  |  |  |  |  |  |  |  |  |  |  |  |  |  |  |  |  |  |  |
| par\_52 | .000 | .000 | .000 | .000 | .000 | .000 | .000 | .000 | .000 | .000 | .000 | .000 | .000 | .000 | .000 | .000 | .000 | .000 | .000 | .000 | .000 | .000 | .000 | .000 | .000 | .000 | .000 | .000 | .000 | .000 | .000 | .000 | .000 | .000 | .000 | .000 | .000 | .000 | .000 | .000 | .000 | .301 | .370 | .357 | .446 | .433 | .320 | .386 | .427 | .450 | .583 | 1.000 |  |  |  |  |  |  |  |  |  |  |  |  |  |  |  |  |  |  |  |  |  |  |  |  |  |  |  |  |  |  |  |  |  |  |  |  |  |  |  |  |  |  |  |  |  |  |  |  |  |  |  |
| par\_53 | .000 | .000 | .000 | .000 | .000 | .000 | .000 | .000 | .000 | .000 | .000 | .000 | .000 | .000 | .000 | .000 | .000 | .000 | .000 | .000 | .000 | .000 | .000 | .000 | .000 | .000 | .000 | .000 | .000 | .000 | .000 | .000 | .000 | .000 | .000 | .000 | .000 | .000 | .000 | .000 | .000 | .339 | .417 | .402 | .503 | .488 | .361 | .435 | .481 | .507 | .657 | .729 | 1.000 |  |  |  |  |  |  |  |  |  |  |  |  |  |  |  |  |  |  |  |  |  |  |  |  |  |  |  |  |  |  |  |  |  |  |  |  |  |  |  |  |  |  |  |  |  |  |  |  |  |  |
| par\_54 | .000 | .000 | .000 | .000 | .000 | .000 | .000 | .000 | .000 | .000 | .000 | .000 | .000 | .000 | .000 | .000 | .000 | .000 | .000 | .000 | .000 | .000 | .000 | .000 | .000 | .000 | .000 | .000 | .000 | .000 | .000 | .000 | .000 | .000 | .000 | .000 | .000 | .000 | .000 | .000 | .000 | .100 | .123 | .118 | .148 | .144 | .106 | .128 | .141 | .149 | .193 | .214 | .242 | 1.000 |  |  |  |  |  |  |  |  |  |  |  |  |  |  |  |  |  |  |  |  |  |  |  |  |  |  |  |  |  |  |  |  |  |  |  |  |  |  |  |  |  |  |  |  |  |  |  |  |  |
| par\_55 | .000 | .000 | .000 | .000 | .000 | .000 | .000 | .000 | .000 | .000 | .000 | .000 | .000 | .000 | .000 | .000 | .000 | .000 | .000 | .000 | .000 | .000 | .000 | .000 | .000 | .000 | .000 | .000 | .000 | .000 | .000 | .000 | .000 | .000 | .000 | .000 | .000 | .000 | .000 | .000 | .000 | .093 | .114 | .110 | .138 | .134 | .099 | .119 | .132 | .139 | .180 | .200 | .225 | .406 | 1.000 |  |  |  |  |  |  |  |  |  |  |  |  |  |  |  |  |  |  |  |  |  |  |  |  |  |  |  |  |  |  |  |  |  |  |  |  |  |  |  |  |  |  |  |  |  |  |  |  |
| par\_56 | .000 | .000 | .000 | .000 | .000 | .000 | .000 | .000 | .000 | .000 | .000 | .000 | .000 | .000 | .000 | .000 | .000 | .000 | .000 | .000 | .000 | .000 | .000 | .000 | .000 | .000 | .000 | .000 | .000 | .000 | .000 | .000 | .000 | .000 | .000 | .000 | .000 | .000 | .000 | .000 | .000 | .124 | .152 | .147 | .184 | .178 | .132 | .159 | .176 | .185 | .240 | .266 | .300 | .541 | .504 | 1.000 |  |  |  |  |  |  |  |  |  |  |  |  |  |  |  |  |  |  |  |  |  |  |  |  |  |  |  |  |  |  |  |  |  |  |  |  |  |  |  |  |  |  |  |  |  |  |  |
| par\_57 | .000 | .000 | .000 | .000 | .000 | .000 | .000 | .000 | .000 | .000 | .000 | .000 | .000 | .000 | .000 | .000 | .000 | .000 | .000 | .000 | .000 | .000 | .000 | .000 | .000 | .000 | .000 | .000 | .000 | .000 | .000 | .000 | .000 | .000 | .000 | .000 | .000 | .000 | .000 | .000 | .000 | .025 | .030 | .029 | .037 | .035 | .015 | .018 | .020 | .021 | .016 | .017 | .020 | .006 | .005 | .007 | 1.000 |  |  |  |  |  |  |  |  |  |  |  |  |  |  |  |  |  |  |  |  |  |  |  |  |  |  |  |  |  |  |  |  |  |  |  |  |  |  |  |  |  |  |  |  |  |  |
| par\_58 | .000 | .000 | .000 | .000 | .000 | .000 | .000 | .000 | .000 | .000 | .000 | .000 | .000 | .000 | .000 | .000 | .000 | .000 | .000 | .000 | .000 | .000 | .000 | .000 | .000 | .000 | .000 | .000 | .000 | .000 | .000 | .000 | .000 | .000 | .000 | .000 | .000 | .000 | .000 | .000 | .000 | .023 | .028 | .027 | .034 | .033 | .014 | .017 | .019 | .020 | .015 | .016 | .018 | .005 | .005 | .007 | .545 | 1.000 |  |  |  |  |  |  |  |  |  |  |  |  |  |  |  |  |  |  |  |  |  |  |  |  |  |  |  |  |  |  |  |  |  |  |  |  |  |  |  |  |  |  |  |  |  |
| par\_59 | .000 | .000 | .000 | .000 | .000 | .000 | .000 | .000 | .000 | .000 | .000 | .000 | .000 | .000 | .000 | .000 | .000 | .000 | .000 | .000 | .000 | .000 | .000 | .000 | .000 | .000 | .000 | .000 | .000 | .000 | .000 | .000 | .000 | .000 | .000 | .000 | .000 | .000 | .000 | .000 | .000 | .022 | .027 | .026 | .032 | .031 | .013 | .016 | .017 | .018 | .014 | .015 | .017 | .005 | .005 | .006 | .513 | .479 | 1.000 |  |  |  |  |  |  |  |  |  |  |  |  |  |  |  |  |  |  |  |  |  |  |  |  |  |  |  |  |  |  |  |  |  |  |  |  |  |  |  |  |  |  |  |  |
| par\_60 | .000 | .000 | .000 | .000 | .000 | .000 | .000 | .000 | .000 | .000 | .000 | .000 | .000 | .000 | .000 | .000 | .000 | .000 | .000 | .000 | .000 | .000 | .000 | .000 | .000 | .000 | .000 | .000 | .000 | .000 | .000 | .000 | .000 | .000 | .000 | .000 | .000 | .000 | .000 | .000 | .000 | .025 | .031 | .030 | .038 | .037 | .015 | .019 | .021 | .022 | .016 | .018 | .020 | .006 | .006 | .007 | .602 | .562 | .529 | 1.000 |  |  |  |  |  |  |  |  |  |  |  |  |  |  |  |  |  |  |  |  |  |  |  |  |  |  |  |  |  |  |  |  |  |  |  |  |  |  |  |  |  |  |  |
| par\_61 | .000 | .000 | .000 | .000 | .000 | .000 | .000 | .000 | .000 | .000 | .000 | .000 | .000 | .000 | .000 | .000 | .000 | .000 | .000 | .000 | .000 | .000 | .000 | .000 | .000 | .000 | .000 | .000 | .000 | .000 | .000 | .000 | .000 | .000 | .000 | .000 | .000 | .000 | .000 | .000 | .000 | .006 | .008 | .007 | .009 | .009 | -.030 | -.037 | -.041 | -.043 | .057 | .064 | .072 | .122 | .114 | .151 | .201 | .188 | .177 | .207 | 1.000 |  |  |  |  |  |  |  |  |  |  |  |  |  |  |  |  |  |  |  |  |  |  |  |  |  |  |  |  |  |  |  |  |  |  |  |  |  |  |  |  |  |  |
| par\_62 | .000 | .000 | .000 | .000 | .000 | .000 | .000 | .000 | .000 | .000 | .000 | .000 | .000 | .000 | .000 | .000 | .000 | .000 | .000 | .000 | .000 | .000 | .000 | .000 | .000 | .000 | .000 | .000 | .000 | .000 | .000 | .000 | .000 | .000 | .000 | .000 | .000 | .000 | .000 | .000 | .000 | .006 | .007 | .007 | .009 | .009 | -.029 | -.035 | -.039 | -.041 | .055 | .061 | .069 | .118 | .110 | .146 | .194 | .181 | .171 | .200 | .717 | 1.000 |  |  |  |  |  |  |  |  |  |  |  |  |  |  |  |  |  |  |  |  |  |  |  |  |  |  |  |  |  |  |  |  |  |  |  |  |  |  |  |  |  |
| par\_63 | .000 | .000 | .000 | .000 | .000 | .000 | .000 | .000 | .000 | .000 | .000 | .000 | .000 | .000 | .000 | .000 | .000 | .000 | .000 | .000 | .000 | .000 | .000 | .000 | .000 | .000 | .000 | .000 | .000 | .000 | .000 | .000 | .000 | .000 | .000 | .000 | .000 | .000 | .000 | .000 | .000 | .006 | .007 | .007 | .009 | .008 | -.029 | -.035 | -.039 | -.041 | .054 | .060 | .068 | .116 | .108 | .144 | .191 | .178 | .168 | .197 | .706 | .872 | 1.000 |  |  |  |  |  |  |  |  |  |  |  |  |  |  |  |  |  |  |  |  |  |  |  |  |  |  |  |  |  |  |  |  |  |  |  |  |  |  |  |  |
| par\_64 | .000 | .000 | .000 | .000 | .000 | .000 | .000 | .000 | .000 | .000 | .000 | .000 | .000 | .000 | .000 | .000 | .000 | .000 | .000 | .000 | .000 | .000 | .000 | .000 | .000 | .000 | .000 | .000 | .000 | .000 | .000 | .000 | .000 | .000 | .000 | .000 | .000 | .000 | .000 | .000 | .000 | .006 | .007 | .007 | .009 | .008 | -.029 | -.035 | -.038 | -.040 | .054 | .060 | .067 | .115 | .107 | .143 | .189 | .177 | .167 | .195 | .739 | .675 | .665 | 1.000 |  |  |  |  |  |  |  |  |  |  |  |  |  |  |  |  |  |  |  |  |  |  |  |  |  |  |  |  |  |  |  |  |  |  |  |  |  |  |  |
| par\_65 | .000 | .000 | .000 | .000 | .000 | .000 | .000 | .000 | .000 | .000 | .000 | .000 | .000 | .000 | .000 | .000 | .000 | .000 | .000 | .000 | .000 | .000 | .000 | .000 | .000 | .000 | .000 | .000 | .000 | .000 | .000 | .000 | .000 | .000 | .000 | .000 | .000 | .000 | .000 | .000 | .000 | .005 | .006 | .006 | .008 | .007 | -.025 | -.030 | -.033 | -.035 | .047 | .052 | .058 | .100 | .093 | .124 | .164 | .153 | .144 | .169 | .607 | .585 | .577 | .572 | 1.000 |  |  |  |  |  |  |  |  |  |  |  |  |  |  |  |  |  |  |  |  |  |  |  |  |  |  |  |  |  |  |  |  |  |  |  |  |  |  |
| par\_66 | .000 | .000 | .000 | .000 | .000 | .000 | .000 | .000 | .000 | .000 | .000 | .000 | .000 | .000 | .000 | .000 | .000 | .000 | .000 | .000 | .000 | .000 | .000 | .000 | .000 | .000 | .000 | .000 | .000 | .000 | .000 | .000 | .000 | .000 | .000 | .000 | .000 | .000 | .000 | .000 | .000 | .020 | .024 | .023 | .029 | .028 | .012 | .014 | .016 | .017 | .013 | .014 | .016 | .005 | .004 | .006 | .468 | .437 | .412 | .483 | .161 | .156 | .153 | .152 | .132 | 1.000 |  |  |  |  |  |  |  |  |  |  |  |  |  |  |  |  |  |  |  |  |  |  |  |  |  |  |  |  |  |  |  |  |  |  |  |  |  |
| par\_67 | .000 | .000 | .000 | .000 | .000 | .000 | .000 | .000 | .000 | .000 | .000 | .000 | .000 | .000 | .000 | .000 | .000 | .000 | .000 | .000 | .000 | .000 | .000 | .000 | .000 | .000 | .000 | .000 | .000 | .000 | .000 | .000 | .000 | .000 | .000 | .000 | .000 | .000 | .000 | .000 | .000 | .004 | .004 | .004 | .005 | .005 | -.018 | -.021 | -.023 | -.025 | .033 | .037 | .041 | .071 | .066 | .088 | .116 | .109 | .102 | .120 | .431 | .415 | .409 | .405 | .591 | .093 | 1.000 |  |  |  |  |  |  |  |  |  |  |  |  |  |  |  |  |  |  |  |  |  |  |  |  |  |  |  |  |  |  |  |  |  |  |  |  |
| par\_68 | .000 | .000 | .000 | .000 | .000 | .000 | .000 | .000 | .000 | .000 | .000 | .000 | .000 | .000 | .000 | .000 | .000 | .000 | .000 | .000 | .000 | .000 | .000 | .000 | .000 | .000 | .000 | .000 | .000 | .000 | .000 | .000 | .000 | .000 | .000 | .000 | .000 | .000 | .000 | .000 | .000 | .004 | .005 | .004 | .005 | .005 | -.018 | -.022 | -.024 | -.025 | .034 | .038 | .043 | .073 | .068 | .090 | .120 | .112 | .105 | .123 | .442 | .426 | .420 | .416 | .578 | .096 | .556 | 1.000 |  |  |  |  |  |  |  |  |  |  |  |  |  |  |  |  |  |  |  |  |  |  |  |  |  |  |  |  |  |  |  |  |  |  |  |
| par\_69 | .000 | .000 | .000 | .000 | .000 | .000 | .000 | .000 | .000 | .000 | .000 | .000 | .000 | .000 | .000 | .000 | .000 | .000 | .000 | .000 | .000 | .000 | .000 | .000 | .000 | .000 | .000 | .000 | .000 | .000 | .000 | .000 | .000 | .000 | .000 | .000 | .000 | .000 | .000 | .000 | .000 | .215 | .264 | .255 | .319 | .310 | .476 | .400 | .442 | .466 | .241 | .268 | .302 | .089 | .083 | .110 | .013 | .012 | .011 | .013 | -.025 | -.025 | -.024 | -.024 | -.021 | .010 | -.015 | -.015 | 1.000 |  |  |  |  |  |  |  |  |  |  |  |  |  |  |  |  |  |  |  |  |  |  |  |  |  |  |  |  |  |  |  |  |  |  |
| par\_70 | .000 | .000 | .000 | -.688 | -.648 | -.834 | -.816 | .000 | .000 | .000 | .000 | .000 | .000 | .000 | .000 | .000 | .000 | .000 | .000 | .000 | .000 | -.046 | .000 | .000 | .000 | .660 | .665 | .158 | .000 | .000 | .000 | .000 | -.033 | .000 | .000 | .000 | .000 | .000 | -.057 | .000 | -.060 | .000 | .000 | .000 | .000 | .000 | .000 | .000 | .000 | .000 | .000 | .000 | .000 | .000 | .000 | .000 | .000 | .000 | .000 | .000 | .000 | .000 | .000 | .000 | .000 | .000 | .000 | .000 | .000 | 1.000 |  |  |  |  |  |  |  |  |  |  |  |  |  |  |  |  |  |  |  |  |  |  |  |  |  |  |  |  |  |  |  |  |  |
| par\_71 | -.001 | .000 | .000 | .000 | .000 | .000 | .000 | -.652 | -.777 | -.805 | .000 | .000 | .000 | .000 | .000 | .000 | .000 | .000 | .000 | .000 | .000 | .000 | .000 | .154 | .000 | .691 | .175 | .703 | .000 | .000 | -.438 | -.144 | .000 | .000 | .000 | .000 | .000 | -.068 | .000 | .000 | .000 | .000 | .000 | .000 | .000 | .000 | .000 | .000 | .000 | .000 | .000 | .000 | .000 | .000 | .000 | .000 | .000 | .000 | .000 | .000 | .000 | .000 | .000 | .000 | .000 | .000 | .000 | .000 | .000 | .094 | 1.000 |  |  |  |  |  |  |  |  |  |  |  |  |  |  |  |  |  |  |  |  |  |  |  |  |  |  |  |  |  |  |  |  |
| par\_72 | -.063 | .000 | -.257 | .000 | .000 | .000 | .000 | .000 | .000 | .000 | -.642 | -.727 | .000 | .000 | .000 | .000 | .000 | .000 | .000 | .000 | .000 | .000 | .000 | -.001 | .000 | .176 | .628 | .635 | .000 | .000 | .000 | .000 | .000 | .000 | .000 | .000 | .000 | .000 | .000 | .000 | .000 | .000 | .000 | .000 | .000 | .000 | .000 | .000 | .000 | .000 | .000 | .000 | .000 | .000 | .000 | .000 | .000 | .000 | .000 | .000 | .000 | .000 | .000 | .000 | .000 | .000 | .000 | .000 | .000 | .095 | .107 | 1.000 |  |  |  |  |  |  |  |  |  |  |  |  |  |  |  |  |  |  |  |  |  |  |  |  |  |  |  |  |  |  |  |
| par\_73 | .001 | .000 | .000 | .000 | .000 | .000 | .000 | .000 | .000 | .000 | .000 | .000 | .000 | .000 | -.541 | -.507 | -.604 | .000 | .000 | .000 | .000 | .025 | -.214 | .001 | -.461 | .000 | .000 | .000 | .000 | .000 | .000 | .000 | .000 | .000 | .000 | .000 | .000 | .000 | .000 | .000 | .000 | .000 | .000 | .000 | .000 | .000 | .000 | .000 | .000 | .000 | .000 | .000 | .000 | .000 | .000 | .000 | .000 | .000 | .000 | .000 | .000 | .000 | .000 | .000 | .000 | .000 | .000 | .000 | .000 | .000 | .000 | .000 | 1.000 |  |  |  |  |  |  |  |  |  |  |  |  |  |  |  |  |  |  |  |  |  |  |  |  |  |  |  |  |  |  |
| par\_74 | .010 | -.198 | .295 | .000 | .000 | .000 | .000 | .000 | .000 | .000 | .000 | .004 | -.583 | -.732 | .000 | .000 | .000 | .000 | .000 | .000 | .000 | .000 | .000 | .000 | .000 | .000 | .000 | .000 | .000 | .000 | .000 | .000 | .000 | .000 | .000 | .000 | .000 | .000 | .000 | .000 | .000 | .000 | .000 | .000 | .000 | .000 | .000 | .000 | .000 | .000 | .000 | .000 | .000 | .000 | .000 | .000 | .000 | .000 | .000 | .000 | .000 | .000 | .000 | .000 | .000 | .000 | .000 | .000 | .000 | .000 | .000 | .000 | .000 | 1.000 |  |  |  |  |  |  |  |  |  |  |  |  |  |  |  |  |  |  |  |  |  |  |  |  |  |  |  |  |  |
| par\_75 | .008 | -.002 | -.002 | .000 | .000 | .000 | .000 | .000 | .000 | .000 | .000 | .001 | .000 | .008 | .000 | .000 | .000 | -.518 | -.508 | -.356 | -.406 | .000 | .036 | .000 | -.001 | .000 | .000 | -.001 | -.270 | -.278 | .000 | .000 | .000 | .046 | .209 | -.310 | .028 | .000 | .000 | .044 | .000 | .000 | .000 | .000 | .000 | .000 | .000 | .000 | .000 | .000 | .000 | .000 | .000 | .000 | .000 | .000 | .000 | .000 | .000 | .000 | .000 | .000 | .000 | .000 | .000 | .000 | .000 | .000 | .000 | .000 | .000 | .000 | .002 | -.001 | 1.000 |  |  |  |  |  |  |  |  |  |  |  |  |  |  |  |  |  |  |  |  |  |  |  |  |  |  |  |  |
| par\_76 | .000 | .000 | .000 | .042 | .040 | .060 | .051 | .000 | .000 | .000 | .000 | .000 | .000 | .000 | .000 | .000 | .000 | .000 | .000 | .000 | .000 | .003 | .000 | .000 | .000 | -.026 | -.026 | .000 | .000 | .000 | .000 | .000 | .150 | .000 | .000 | .000 | .000 | .000 | .489 | .000 | .415 | .000 | .000 | .000 | .000 | .000 | .000 | .000 | .000 | .000 | .000 | .000 | .000 | .000 | .000 | .000 | .000 | .000 | .000 | .000 | .000 | .000 | .000 | .000 | .000 | .000 | .000 | .000 | .000 | -.048 | .000 | .000 | .000 | .000 | .000 | 1.000 |  |  |  |  |  |  |  |  |  |  |  |  |  |  |  |  |  |  |  |  |  |  |  |  |  |  |  |
| par\_77 | .000 | .000 | .000 | -.028 | -.002 | .040 | .022 | .000 | .000 | .000 | .000 | .000 | .000 | .000 | .000 | .000 | .000 | .000 | .000 | .000 | .000 | .001 | .000 | .000 | .000 | -.014 | -.014 | .000 | .000 | .000 | .000 | .000 | .588 | .000 | .000 | .000 | .000 | .000 | .191 | .000 | .419 | .000 | .000 | .000 | .000 | .000 | .000 | .000 | .000 | .000 | .000 | .000 | .000 | .000 | .000 | .000 | .000 | .000 | .000 | .000 | .000 | .000 | .000 | .000 | .000 | .000 | .000 | .000 | .000 | -.024 | .000 | .000 | .000 | .000 | .000 | .098 | 1.000 |  |  |  |  |  |  |  |  |  |  |  |  |  |  |  |  |  |  |  |  |  |  |  |  |  |  |
| par\_78 | .000 | .000 | .000 | .005 | -.026 | .040 | .025 | .000 | .000 | .000 | .000 | .000 | .000 | .000 | .000 | .000 | .000 | .000 | .000 | .000 | .000 | .002 | .000 | .000 | .000 | -.014 | -.015 | .000 | .000 | .000 | .000 | .000 | .586 | .000 | .000 | .000 | .000 | .000 | .492 | .000 | .232 | .000 | .000 | .000 | .000 | .000 | .000 | .000 | .000 | .000 | .000 | .000 | .000 | .000 | .000 | .000 | .000 | .000 | .000 | .000 | .000 | .000 | .000 | .000 | .000 | .000 | .000 | .000 | .000 | -.026 | .000 | .000 | .000 | .000 | .000 | .139 | .212 | 1.000 |  |  |  |  |  |  |  |  |  |  |  |  |  |  |  |  |  |  |  |  |  |  |  |  |  |
| par\_79 | .000 | .000 | .000 | .000 | .000 | -.143 | .038 | .000 | .000 | .000 | .000 | .000 | .000 | .000 | .000 | .000 | .000 | .000 | .000 | .000 | .000 | .000 | .000 | .000 | .000 | .034 | .034 | .000 | .000 | .000 | .000 | .000 | -.131 | .000 | .000 | .000 | .000 | .000 | -.089 | .000 | -.099 | .000 | .000 | .000 | .000 | .000 | .000 | .000 | .000 | .000 | .000 | .000 | .000 | .000 | .000 | .000 | .000 | .000 | .000 | .000 | .000 | .000 | .000 | .000 | .000 | .000 | .000 | .000 | .000 | .035 | .000 | .000 | .000 | .000 | .000 | -.048 | -.111 | -.094 | 1.000 |  |  |  |  |  |  |  |  |  |  |  |  |  |  |  |  |  |  |  |  |  |  |  |  |
| par\_80 | .000 | .000 | .000 | .000 | .000 | .079 | -.079 | .000 | .000 | .000 | .000 | .000 | .000 | .000 | .000 | .000 | .000 | .000 | .000 | .000 | .000 | .000 | .000 | .000 | .000 | -.010 | -.010 | .000 | .000 | .000 | .000 | .000 | .039 | .000 | .000 | .000 | .000 | .000 | .027 | .000 | .030 | .000 | .000 | .000 | .000 | .000 | .000 | .000 | .000 | .000 | .000 | .000 | .000 | .000 | .000 | .000 | .000 | .000 | .000 | .000 | .000 | .000 | .000 | .000 | .000 | .000 | .000 | .000 | .000 | -.010 | .000 | .000 | .000 | .000 | .000 | .014 | .033 | .028 | -.567 | 1.000 |  |  |  |  |  |  |  |  |  |  |  |  |  |  |  |  |  |  |  |  |  |  |  |
| par\_81 | .001 | .000 | .000 | .000 | .000 | .000 | .000 | .104 | .126 | .137 | .000 | .000 | .000 | .000 | .000 | .000 | .000 | .000 | .000 | .000 | .000 | .000 | .000 | -.025 | .000 | -.068 | .000 | -.069 | .000 | .000 | .087 | .634 | .000 | .000 | .000 | .000 | .000 | .319 | .000 | .000 | .000 | .000 | .000 | .000 | .000 | .000 | .000 | .000 | .000 | .000 | .000 | .000 | .000 | .000 | .000 | .000 | .000 | .000 | .000 | .000 | .000 | .000 | .000 | .000 | .000 | .000 | .000 | .000 | .000 | .000 | -.122 | .000 | .000 | .000 | .000 | .000 | .000 | .000 | .000 | .000 | 1.000 |  |  |  |  |  |  |  |  |  |  |  |  |  |  |  |  |  |  |  |  |  |  |
| par\_82 | .002 | .000 | .000 | .000 | .000 | .000 | .000 | -.050 | .095 | .114 | .000 | .000 | .000 | .000 | .000 | .000 | .000 | .000 | .000 | .000 | .000 | .000 | .000 | -.019 | .000 | -.045 | .000 | -.045 | .000 | .000 | .070 | .651 | .000 | .000 | .000 | .000 | .000 | .001 | .000 | .000 | .000 | .000 | .000 | .000 | .000 | .000 | .000 | .000 | .000 | .000 | .000 | .000 | .000 | .000 | .000 | .000 | .000 | .000 | .000 | .000 | .000 | .000 | .000 | .000 | .000 | .000 | .000 | .000 | .000 | .000 | -.085 | .000 | .000 | .000 | .000 | .000 | .000 | .000 | .000 | .000 | .255 | 1.000 |  |  |  |  |  |  |  |  |  |  |  |  |  |  |  |  |  |  |  |  |  |
| par\_83 | .005 | .000 | .000 | .000 | .000 | .000 | .000 | .000 | -.158 | .046 | .000 | .000 | .000 | .000 | .000 | .000 | .000 | .000 | .000 | .000 | .000 | .000 | .000 | -.003 | .000 | .016 | .000 | .016 | .000 | .000 | -.003 | -.029 | .000 | .000 | .000 | .000 | .000 | -.007 | .000 | .000 | .000 | .000 | .000 | .000 | .000 | .000 | .000 | .000 | .000 | .000 | .000 | .000 | .000 | .000 | .000 | .000 | .000 | .000 | .000 | .000 | .000 | .000 | .000 | .000 | .000 | .000 | .000 | .000 | .000 | .000 | .015 | .000 | .000 | .000 | -.001 | .000 | .000 | .000 | .000 | .000 | -.015 | -.037 | 1.000 |  |  |  |  |  |  |  |  |  |  |  |  |  |  |  |  |  |  |  |  |
| par\_84 | .016 | .000 | .000 | .000 | .000 | .000 | .000 | .001 | .025 | -.212 | .000 | .000 | .000 | .000 | .000 | .000 | .000 | .000 | .000 | .000 | .000 | .000 | .001 | -.009 | .000 | .050 | .000 | .051 | .000 | .000 | -.008 | -.093 | .000 | .000 | .000 | .000 | .000 | -.022 | .000 | .000 | .000 | .000 | .000 | .000 | .000 | .000 | .000 | .000 | .000 | .000 | .000 | .000 | .000 | .000 | .000 | .000 | .000 | .000 | .000 | .000 | .000 | .000 | .000 | .000 | .000 | .000 | .000 | .000 | .000 | .000 | .047 | .000 | .000 | .000 | -.003 | .000 | .000 | .000 | .000 | .000 | -.049 | -.116 | -.257 | 1.000 |  |  |  |  |  |  |  |  |  |  |  |  |  |  |  |  |  |  |  |
| par\_85 | .014 | .001 | .060 | .000 | .000 | .000 | .000 | .000 | .000 | .000 | .146 | .219 | .000 | .000 | .000 | .000 | .000 | .000 | .000 | .000 | .000 | .000 | .000 | .002 | .000 | .000 | -.079 | -.080 | .000 | .000 | .000 | .000 | .000 | .000 | .000 | .000 | .000 | .000 | .000 | .000 | .000 | .000 | .000 | .000 | .000 | .000 | .000 | .000 | .000 | .000 | .000 | .000 | .000 | .000 | .000 | .000 | .000 | .000 | .000 | .000 | .000 | .000 | .000 | .000 | .000 | .000 | .000 | .000 | .000 | .000 | .000 | -.162 | .000 | .001 | .000 | .000 | .000 | .000 | .000 | .000 | .000 | .000 | .000 | .000 | 1.000 |  |  |  |  |  |  |  |  |  |  |  |  |  |  |  |  |  |  |
| par\_86 | -.002 | .001 | .001 | .000 | .000 | .000 | .000 | .000 | .000 | .000 | -.170 | .111 | .000 | .000 | .000 | .000 | .000 | .000 | .000 | .000 | .000 | .000 | .000 | .003 | .000 | .000 | -.011 | -.011 | .000 | .000 | .000 | .000 | .000 | .000 | .000 | .000 | .000 | .000 | .000 | .000 | .000 | .000 | .000 | .000 | .000 | .000 | .000 | .000 | .000 | .000 | .000 | .000 | .000 | .000 | .000 | .000 | .000 | .000 | .000 | .000 | .000 | .000 | .000 | .000 | .000 | .000 | .000 | .000 | .000 | .000 | .000 | -.012 | .000 | .001 | .000 | .000 | .000 | .000 | .000 | .000 | .000 | .000 | .000 | .000 | .020 | 1.000 |  |  |  |  |  |  |  |  |  |  |  |  |  |  |  |  |  |
| par\_87 | .017 | -.009 | -.009 | .000 | .000 | .000 | .000 | .000 | .000 | .000 | .031 | -.357 | .000 | .000 | .000 | .000 | .000 | .000 | .000 | .000 | .000 | .000 | .000 | -.025 | .000 | .000 | .095 | .096 | .000 | .000 | .000 | .000 | .000 | .000 | .000 | .000 | .000 | .000 | .000 | .000 | .000 | .000 | .000 | .000 | .000 | .000 | .000 | .000 | .000 | .000 | .000 | .000 | .000 | .000 | .000 | .000 | .000 | .000 | .000 | .000 | .000 | .000 | .000 | .000 | .000 | .000 | .000 | .000 | .000 | .000 | .000 | .106 | .000 | -.012 | -.002 | .000 | .000 | .000 | .000 | .000 | .000 | .000 | .000 | .000 | -.177 | -.330 | 1.000 |  |  |  |  |  |  |  |  |  |  |  |  |  |  |  |  |
| par\_88 | .005 | .081 | -.184 | .000 | .000 | .000 | .000 | .000 | .000 | .000 | .000 | .000 | .246 | .437 | .000 | .000 | .000 | .000 | .000 | .000 | .000 | .000 | .000 | .000 | .000 | .000 | .000 | .000 | .000 | .000 | .000 | .000 | .000 | .000 | .000 | .000 | .000 | .000 | .000 | .000 | .000 | .000 | .000 | .000 | .000 | .000 | .000 | .000 | .000 | .000 | .000 | .000 | .000 | .000 | .000 | .000 | .000 | .000 | .000 | .000 | .000 | .000 | .000 | .000 | .000 | .000 | .000 | .000 | .000 | .000 | .000 | .000 | .000 | -.345 | .002 | .000 | .000 | .000 | .000 | .000 | .000 | .000 | .000 | .000 | .000 | .000 | .000 | 1.000 |  |  |  |  |  |  |  |  |  |  |  |  |  |  |  |
| par\_89 | .004 | .001 | -.024 | .000 | .000 | .000 | .000 | .000 | .000 | .000 | .000 | .000 | -.225 | .136 | .000 | .000 | .000 | .000 | .000 | .000 | .000 | .000 | .000 | .000 | .000 | .000 | .000 | .000 | .000 | .000 | .000 | .000 | .000 | .000 | .000 | .000 | .000 | .000 | .000 | .000 | .000 | .000 | .000 | .000 | .000 | .000 | .000 | .000 | .000 | .000 | .000 | .000 | .000 | .000 | .000 | .000 | .000 | .000 | .000 | .000 | .000 | .000 | .000 | .000 | .000 | .000 | .000 | .000 | .000 | .000 | .000 | .000 | .000 | -.025 | .002 | .000 | .000 | .000 | .000 | .000 | .000 | .000 | .000 | .000 | .000 | .000 | .000 | .038 | 1.000 |  |  |  |  |  |  |  |  |  |  |  |  |  |  |
| par\_90 | -.035 | -.006 | .213 | .000 | .000 | .000 | .000 | .000 | .000 | .000 | .000 | .000 | -.028 | -.589 | .000 | .000 | .000 | .000 | .000 | .000 | .000 | .000 | .000 | .000 | .000 | .000 | .000 | .000 | .000 | .000 | .000 | .000 | .000 | .000 | .000 | .000 | .000 | .000 | .000 | .000 | .000 | .000 | .000 | .000 | .000 | .000 | .000 | .000 | .000 | .000 | .000 | .000 | .000 | .000 | .000 | .000 | .000 | .000 | .000 | .000 | .000 | .000 | .000 | .000 | .000 | .000 | .000 | .000 | .000 | .000 | .000 | .000 | .000 | .230 | -.014 | .000 | .000 | .000 | .000 | .000 | .000 | .000 | .000 | .000 | .000 | .000 | .000 | -.341 | -.254 | 1.000 |  |  |  |  |  |  |  |  |  |  |  |  |  |
| par\_91 | .000 | .000 | .000 | .000 | .000 | .000 | .000 | .000 | .000 | .000 | .000 | .000 | .000 | .000 | .243 | .225 | .286 | .000 | .000 | .000 | .000 | -.012 | .093 | .000 | .203 | .000 | .000 | .000 | .000 | .000 | .000 | .000 | .000 | .000 | .000 | .000 | .000 | .000 | .000 | .000 | .000 | .000 | .000 | .000 | .000 | .000 | .000 | .000 | .000 | .000 | .000 | .000 | .000 | .000 | .000 | .000 | .000 | .000 | .000 | .000 | .000 | .000 | .000 | .000 | .000 | .000 | .000 | .000 | .000 | .000 | .000 | .000 | -.259 | .000 | -.004 | .000 | .000 | .000 | .000 | .000 | .000 | .000 | .000 | .000 | .000 | .000 | .000 | .000 | .000 | .000 | 1.000 |  |  |  |  |  |  |  |  |  |  |  |  |
| par\_92 | .000 | .000 | .000 | .000 | .000 | .000 | .000 | .000 | .000 | .000 | .000 | .000 | .000 | .000 | -.224 | -.008 | .006 | .000 | .000 | .000 | .000 | .003 | -.005 | .000 | -.009 | .000 | .000 | .000 | .000 | .000 | .000 | .000 | .000 | .000 | .000 | .000 | .000 | .000 | .000 | .000 | .000 | .000 | .000 | .000 | .000 | .000 | .000 | .000 | .000 | .000 | .000 | .000 | .000 | .000 | .000 | .000 | .000 | .000 | .000 | .000 | .000 | .000 | .000 | .000 | .000 | .000 | .000 | .000 | .000 | .000 | .000 | .000 | .035 | .000 | -.002 | .000 | .000 | .000 | .000 | .000 | .000 | .000 | .000 | .000 | .000 | .000 | .000 | .000 | .000 | .000 | -.062 | 1.000 |  |  |  |  |  |  |  |  |  |  |  |
| par\_93 | .000 | .000 | .000 | .000 | .000 | .000 | .000 | .000 | .000 | .000 | .000 | .000 | .000 | .000 | -.004 | -.199 | .005 | .000 | .000 | .000 | .000 | .002 | -.004 | .000 | -.007 | .000 | .000 | .000 | .000 | .000 | .000 | .000 | .000 | .000 | .000 | .000 | .000 | .000 | .000 | .000 | .000 | .000 | .000 | .000 | .000 | .000 | .000 | .000 | .000 | .000 | .000 | .000 | .000 | .000 | .000 | .000 | .000 | .000 | .000 | .000 | .000 | .000 | .000 | .000 | .000 | .000 | .000 | .000 | .000 | .000 | .000 | .000 | .026 | .000 | -.002 | .000 | .000 | .000 | .000 | .000 | .000 | .000 | .000 | .000 | .000 | .000 | .000 | .000 | .000 | .000 | -.046 | -.029 | 1.000 |  |  |  |  |  |  |  |  |  |  |
| par\_94 | .000 | .000 | .000 | .000 | .000 | .000 | .000 | .000 | .000 | .000 | .000 | .000 | .000 | .000 | -.012 | -.016 | -.300 | .000 | .000 | .000 | .000 | .006 | -.011 | .000 | -.018 | .000 | .000 | .000 | .000 | .000 | .000 | .000 | .000 | .000 | .000 | .000 | .000 | .000 | .000 | .000 | .000 | .000 | .000 | .000 | .000 | .000 | .000 | .000 | .000 | .000 | .000 | .000 | .000 | .000 | .000 | .000 | .000 | .000 | .000 | .000 | .000 | .000 | .000 | .000 | .000 | .000 | .000 | .000 | .000 | .000 | .000 | .000 | .073 | .000 | -.005 | .000 | .000 | .000 | .000 | .000 | .000 | .000 | .000 | .000 | .000 | .000 | .000 | .000 | .000 | .000 | -.127 | -.081 | -.061 | 1.000 |  |  |  |  |  |  |  |  |  |
| par\_95 | -.022 | -.046 | .000 | .000 | .000 | .000 | .000 | .000 | .000 | .000 | .000 | .000 | .000 | .000 | .000 | .000 | .000 | .496 | .486 | .159 | .342 | .000 | -.076 | .037 | .000 | .000 | .000 | .000 | .227 | .234 | .000 | .000 | .000 | -.099 | -.452 | .688 | -.060 | .000 | .000 | -.095 | .000 | .000 | .000 | .000 | .000 | .000 | .000 | .000 | .000 | .000 | .000 | .000 | .000 | .000 | .000 | .000 | .000 | .000 | .000 | .000 | .000 | .000 | .000 | .000 | .000 | .000 | .000 | .000 | .000 | .000 | .000 | .000 | .000 | .000 | -.355 | .000 | .000 | .000 | .000 | .000 | .000 | .000 | .000 | .000 | .000 | .000 | .000 | .000 | .000 | .000 | .000 | .000 | .000 | .000 | 1.000 |  |  |  |  |  |  |  |  |
| par\_96 | .015 | .031 | .000 | .000 | .000 | .000 | .000 | .000 | .000 | .000 | .000 | .000 | .000 | .000 | .000 | .000 | .000 | -.446 | -.387 | .000 | -.160 | .000 | .052 | -.025 | .000 | .000 | .000 | .000 | -.106 | -.109 | .000 | .000 | .000 | .013 | .865 | -.459 | .008 | .000 | .000 | .013 | .000 | .000 | .000 | .000 | .000 | .000 | .000 | .000 | .000 | .000 | .000 | .000 | .000 | .000 | .000 | .000 | .000 | .000 | .000 | .000 | .000 | .000 | .000 | .000 | .000 | .000 | .000 | .000 | .000 | .000 | .000 | .000 | .000 | .000 | .193 | .000 | .000 | .000 | .000 | .000 | .000 | .000 | .000 | .000 | .000 | .000 | .000 | .000 | .000 | .000 | .000 | .000 | .000 | .000 | -.417 | 1.000 |  |  |  |  |  |  |  |
| par\_97 | .014 | .029 | .000 | .000 | .000 | .000 | .000 | .000 | .000 | .000 | .000 | .000 | .000 | .000 | .000 | .000 | .000 | -.375 | -.428 | .000 | -.152 | .000 | .049 | -.024 | .000 | .000 | .000 | .000 | -.101 | -.104 | .000 | .000 | .000 | .012 | .861 | -.435 | .007 | .000 | .000 | .012 | .000 | .000 | .000 | .000 | .000 | .000 | .000 | .000 | .000 | .000 | .000 | .000 | .000 | .000 | .000 | .000 | .000 | .000 | .000 | .000 | .000 | .000 | .000 | .000 | .000 | .000 | .000 | .000 | .000 | .000 | .000 | .000 | .000 | .000 | .183 | .000 | .000 | .000 | .000 | .000 | .000 | .000 | .000 | .000 | .000 | .000 | .000 | .000 | .000 | .000 | .000 | .000 | .000 | .000 | -.396 | .617 | 1.000 |  |  |  |  |  |  |
| par\_98 | -.008 | -.017 | .000 | .000 | .000 | .000 | .000 | .000 | .000 | .000 | .000 | .000 | .000 | .000 | .000 | .000 | .000 | .295 | .289 | -.206 | .185 | .000 | -.028 | .014 | .000 | .000 | .000 | .000 | .123 | .127 | .000 | .000 | .000 | -.081 | -.368 | .663 | -.049 | .000 | .000 | -.077 | .000 | .000 | .000 | .000 | .000 | .000 | .000 | .000 | .000 | .000 | .000 | .000 | .000 | .000 | .000 | .000 | .000 | .000 | .000 | .000 | .000 | .000 | .000 | .000 | .000 | .000 | .000 | .000 | .000 | .000 | .000 | .000 | .000 | .000 | -.170 | .000 | .000 | .000 | .000 | .000 | .000 | .000 | .000 | .000 | .000 | .000 | .000 | .000 | .000 | .000 | .000 | .000 | .000 | .000 | .351 | -.340 | -.322 | 1.000 |  |  |  |  |  |
| par\_99 | .004 | .009 | .000 | .000 | .000 | .000 | .000 | .000 | .000 | .000 | .000 | .000 | .000 | .000 | .000 | .000 | .000 | -.057 | -.056 | .000 | -.195 | .000 | .015 | -.007 | .000 | .000 | .000 | .000 | -.101 | -.098 | .000 | .000 | .000 | .537 | .017 | -.132 | .233 | .000 | .000 | .567 | .000 | .000 | .000 | .000 | .000 | .000 | .000 | .000 | .000 | .000 | .000 | .000 | .000 | .000 | .000 | .000 | .000 | .000 | .000 | .000 | .000 | .000 | .000 | .000 | .000 | .000 | .000 | .000 | .000 | .000 | .000 | .000 | .000 | .000 | .055 | .000 | .000 | .000 | .000 | .000 | .000 | .000 | .000 | .000 | .000 | .000 | .000 | .000 | .000 | .000 | .000 | .000 | .000 | .000 | -.120 | .016 | .015 | -.097 | 1.000 |  |  |  |  |
| par\_100 | .000 | .000 | .000 | .000 | .000 | .000 | .000 | .000 | .000 | .000 | .000 | .000 | .000 | .000 | -.003 | -.004 | .003 | .000 | .000 | .000 | .000 | .001 | -.003 | .000 | -.170 | .000 | .000 | .000 | .000 | .000 | .000 | .000 | .000 | .000 | .000 | .000 | .000 | .000 | .000 | .000 | .000 | .000 | .000 | .000 | .000 | .000 | .000 | .000 | .000 | .000 | .000 | .000 | .000 | .000 | .000 | .000 | .000 | .000 | .000 | .000 | .000 | .000 | .000 | .000 | .000 | .000 | .000 | .000 | .000 | .000 | .000 | .000 | .018 | .000 | -.001 | .000 | .000 | .000 | .000 | .000 | .000 | .000 | .000 | .000 | .000 | .000 | .000 | .000 | .000 | .000 | -.032 | -.020 | -.015 | -.042 | .000 | .000 | .000 | .000 | .000 | 1.000 |  |  |  |
| par\_101 | .002 | .003 | .000 | .000 | .000 | .000 | .000 | .000 | .000 | .000 | .000 | .000 | .000 | .000 | .000 | .000 | .000 | -.021 | -.021 | .000 | -.057 | .000 | .005 | -.003 | .000 | .000 | .000 | .000 | -.110 | -.055 | .000 | .000 | .000 | .248 | .006 | -.049 | .561 | .000 | .000 | .561 | .000 | .000 | .000 | .000 | .000 | .000 | .000 | .000 | .000 | .000 | .000 | .000 | .000 | .000 | .000 | .000 | .000 | .000 | .000 | .000 | .000 | .000 | .000 | .000 | .000 | .000 | .000 | .000 | .000 | .000 | .000 | .000 | .000 | .000 | .021 | .000 | .000 | .000 | .000 | .000 | .000 | .000 | .000 | .000 | .000 | .000 | .000 | .000 | .000 | .000 | .000 | .000 | .000 | .000 | -.044 | .006 | .006 | -.036 | .191 | .000 | 1.000 |  |  |
| par\_102 | .002 | .004 | .000 | .000 | .000 | .000 | .000 | .000 | .000 | .000 | .000 | .000 | .000 | .000 | .000 | .000 | .000 | -.023 | -.022 | .000 | -.057 | .000 | .006 | -.003 | .000 | .000 | .000 | .000 | -.057 | -.114 | .000 | .000 | .000 | .530 | .007 | -.052 | .561 | .000 | .000 | .230 | .000 | .000 | .000 | .000 | .000 | .000 | .000 | .000 | .000 | .000 | .000 | .000 | .000 | .000 | .000 | .000 | .000 | .000 | .000 | .000 | .000 | .000 | .000 | .000 | .000 | .000 | .000 | .000 | .000 | .000 | .000 | .000 | .000 | .000 | .022 | .000 | .000 | .000 | .000 | .000 | .000 | .000 | .000 | .000 | .000 | .000 | .000 | .000 | .000 | .000 | .000 | .000 | .000 | .000 | -.048 | .006 | .006 | -.039 | .169 | .000 | .188 | 1.000 |  |
| par\_103 | .001 | .000 | .000 | .000 | .000 | .000 | .000 | .025 | .021 | .026 | .000 | .000 | .000 | .000 | .000 | .000 | .000 | .000 | .000 | .000 | .000 | .000 | .000 | -.004 | .000 | -.010 | .000 | -.010 | .000 | .000 | -.073 | -.002 | .000 | .000 | .000 | .000 | .000 | .356 | .000 | .000 | .000 | .000 | .000 | .000 | .000 | .000 | .000 | .000 | .000 | .000 | .000 | .000 | .000 | .000 | .000 | .000 | .000 | .000 | .000 | .000 | .000 | .000 | .000 | .000 | .000 | .000 | .000 | .000 | .000 | .000 | -.018 | .000 | .000 | .000 | .000 | .000 | .000 | .000 | .000 | .000 | .050 | -.004 | -.009 | -.028 | .000 | .000 | .000 | .000 | .000 | .000 | .000 | .000 | .000 | .000 | .000 | .000 | .000 | .000 | .000 | .000 | .000 | .000 | 1.000 |

## Model Fit Summary

## CMIN

| Model | NPAR | CMIN | DF | P | CMIN/DF |
| --- | --- | --- | --- | --- | --- |
| Default model | 103 | 647.135 | 331 | .000 | 1.955 |
| Saturated model | 434 | .000 | 0 |  |  |
| Independence model | 56 | 4437.454 | 378 | .000 | 11.739 |

## Baseline Comparisons

| Model | NFI Delta1 | RFI rho1 | IFI Delta2 | TLI rho2 | CFI |
| --- | --- | --- | --- | --- | --- |
| Default model | .854 | .833 | .923 | .911 | .922 |
| Saturated model | 1.000 |  | 1.000 |  | 1.000 |
| Independence model | .000 | .000 | .000 | .000 | .000 |

## Parsimony-Adjusted Measures

| Model | PRATIO | PNFI | PCFI |
| --- | --- | --- | --- |
| Default model | .876 | .748 | .807 |
| Saturated model | .000 | .000 | .000 |
| Independence model | 1.000 | .000 | .000 |

## NCP

| Model | NCP | LO 90 | HI 90 |
| --- | --- | --- | --- |
| Default model | 316.135 | 247.800 | 392.261 |
| Saturated model | .000 | .000 | .000 |
| Independence model | 4059.454 | 3848.595 | 4277.610 |

## FMIN

| Model | FMIN | F0 | LO 90 | HI 90 |
| --- | --- | --- | --- | --- |
| Default model | 2.838 | 1.387 | 1.087 | 1.720 |
| Saturated model | .000 | .000 | .000 | .000 |
| Independence model | 19.463 | 17.805 | 16.880 | 18.761 |

## RMSEA

| Model | RMSEA | LO 90 | HI 90 | PCLOSE |
| --- | --- | --- | --- | --- |
| Default model | .065 | .057 | .072 | .001 |
| Independence model | .217 | .211 | .223 | .000 |

## AIC

| Model | AIC | BCC | BIC | CAIC |
| --- | --- | --- | --- | --- |
| Default model | 853.135 | 883.155 |  |  |
| Saturated model | 868.000 | 994.492 |  |  |
| Independence model | 4549.454 | 4565.776 |  |  |

## ECVI

| Model | ECVI | LO 90 | HI 90 | MECVI |
| --- | --- | --- | --- | --- |
| Default model | 3.742 | 3.442 | 4.076 | 3.873 |
| Saturated model | 3.807 | 3.807 | 3.807 | 4.362 |
| Independence model | 19.954 | 19.029 | 20.911 | 20.025 |

## HOELTER

| Model | HOELTER .05 | HOELTER .01 |
| --- | --- | --- |
| Default model | 132 | 139 |
| Independence model | 22 | 23 |

## Execution time summary

|  |  |
| --- | --- |
| Minimization: | .034 |
| Miscellaneous: | .679 |
| Bootstrap: | .000 |
| Total: | .713 |
